# Chloroplast PetD protein: evidence for SRP/Alb3-dependent insertion into the thylakoid membrane

**DOI:** 10.1186/s12870-017-1176-2

**Published:** 2017-11-21

**Authors:** Jarosław Króliczewski, Rafał Bartoszewski, Bożena Króliczewska

**Affiliations:** 1 Faculty of Biotechnology, University of Wrocław, Fryderyka Joliot-Curie 14a, 50-383 Wrocław, Poland; 2 Department of Biology and Pharmaceutical Botany Medical University of Gdańsk, Hallera 107, 80-416 Gdansk, Poland; 3 Department of Animal Physiology and Biostructure, Faculty of Veterinary Medicine Wroclaw University of Environmental and Life Sciences, C.K Norwida 31, 50-375 Wrocław, Poland

**Keywords:** Cytochrome *b*_*6*_*f* complex, Thylakoid protein import, PetD, cpSECY, cpSRP54, ALB3

## Abstract

**Background:**

In thylakoid membrane, each monomer of the dimeric complex of cytochrome *b*_*6*_ *f* is comprised of eight subunits that are both nucleus- and plastid-encoded. Proper cytochrome *b*_*6*_ *f* complex integration into the thylakoid membrane requires numerous regulatory factors for coordinated transport, insertion and assembly of the subunits. Although, the chloroplast-encoded cytochrome *b*_*6*_ *f* subunit IV (PetD) consists of three transmembrane helices, the signal and the mechanism of protein integration into the thylakoid membrane have not been identified.

**Results:**

Here, we demonstrate that the native PetD subunit cannot incorporate into the thylakoid membranes spontaneously, but that proper integration occurs through the post-translational signal recognition particle (SRP) pathway. Furthermore, we show that PetD insertion into thylakoid membrane involves the coordinated action of cpFTSY, cpSRP54 and ALB3 insertase.

**Conclusions:**

PetD subunit integration into the thylakoid membrane is a post-translational and an SRP-dependent process that requires the formation of the cpSRP-cpFtsY-ALB3-PetD complex. This data provides a new insight into the molecular mechanisms by which membrane proteins integration into the thylakoid membrane is accomplished and is not limited to PetD.

**Electronic supplementary material:**

The online version of this article (10.1186/s12870-017-1176-2) contains supplementary material, which is available to authorized users.

## Background

The 220-kDa multiprotein cytochrome *b*_*6*_ *f* complex located in the thylakoid membrane provides the electronic connection between the photosystem I (PSI) and II (PSII) reaction centers in the electron transport chain of oxygenic photosynthesis [1, 2]. The cytochrome *b*_*6*_ *f* complex is composed of four major subunits, cytochrome *f* (PetA), a Rieske-type iron–sulfur protein, cytochrome *b*_6_ (PetB), and the chloroplast-encoded subunit IV (PetD), all of which are required for the full catalytic activity of the complex [3]. Furthermore, up to four additional small subunits may also be present in isolated *b*_*6*_ *f* complex preparations [4].

The chloroplast oligomeric complexes that form the photosynthetic electron transfer chain of the thylakoid membrane of higher plants (including cytochrome *b*_*6*_ *f*, PSI, PSII and ATPase) consist of a patchwork of nuclear- and chloroplast-encoded components. Hence, integration of these complexes into the thylakoid membrane requires the assembly of subunits of both chloroplast and nuclear origin and relies on the coordinated action of numerous regulatory factors [5, 6]. However, the majority of plastid proteins are nuclear-encoded and must be imported within these organelles. Targeting of nuclear-encoded proteins to plastid compartments such as the inner envelope membrane, the stroma, and the thylakoid membrane is strictly dependent on the presence of a cleavable transit sequence in the precursor N-terminal region. However, the mechanisms underlying the biosynthesis as well as the targeting to plastid compartments of the chloroplast-encoded proteins, is less understood.

The chloroplast encoded proteins can be imported into or across the thylakoid membrane by one of four independent precursor-specific transport pathways that are categorized as the spontaneous (unassisted) pathway, the signal recognition particle (SRP) pathway, the secretory pathway (Sec) that requires protein substrates to be in an unfolded state for transport, and the twin-arginine translocase-dependent pathway (ΔpH/Tat) that protein substrates can be transported in a folded state, allowing the transport of proteins that fold too quickly or tightly for the Sec pathway. Interestingly, although the Tat pathway post-translationally transports folded proteins mainly across the cytoplasmic membrane, the Tat machinery mediates the insertion of Rieske iron-sulfur protein (a member of cytochrome *b*_*6*_ *f* complex) into the thylakoid membrane in plants [7–9]. Hence, incorporation into the thylakoid membrane for some proteins occurs post-translationally, while others fold and integrate co-translationally such as PetB, PsaA, PsbB, PsbC, PsbD, and PetA [10–12]. The results, however, for PetA incorporation into the thylakoid membrane are inconsistent [12, 13].

**Fig. 1:**
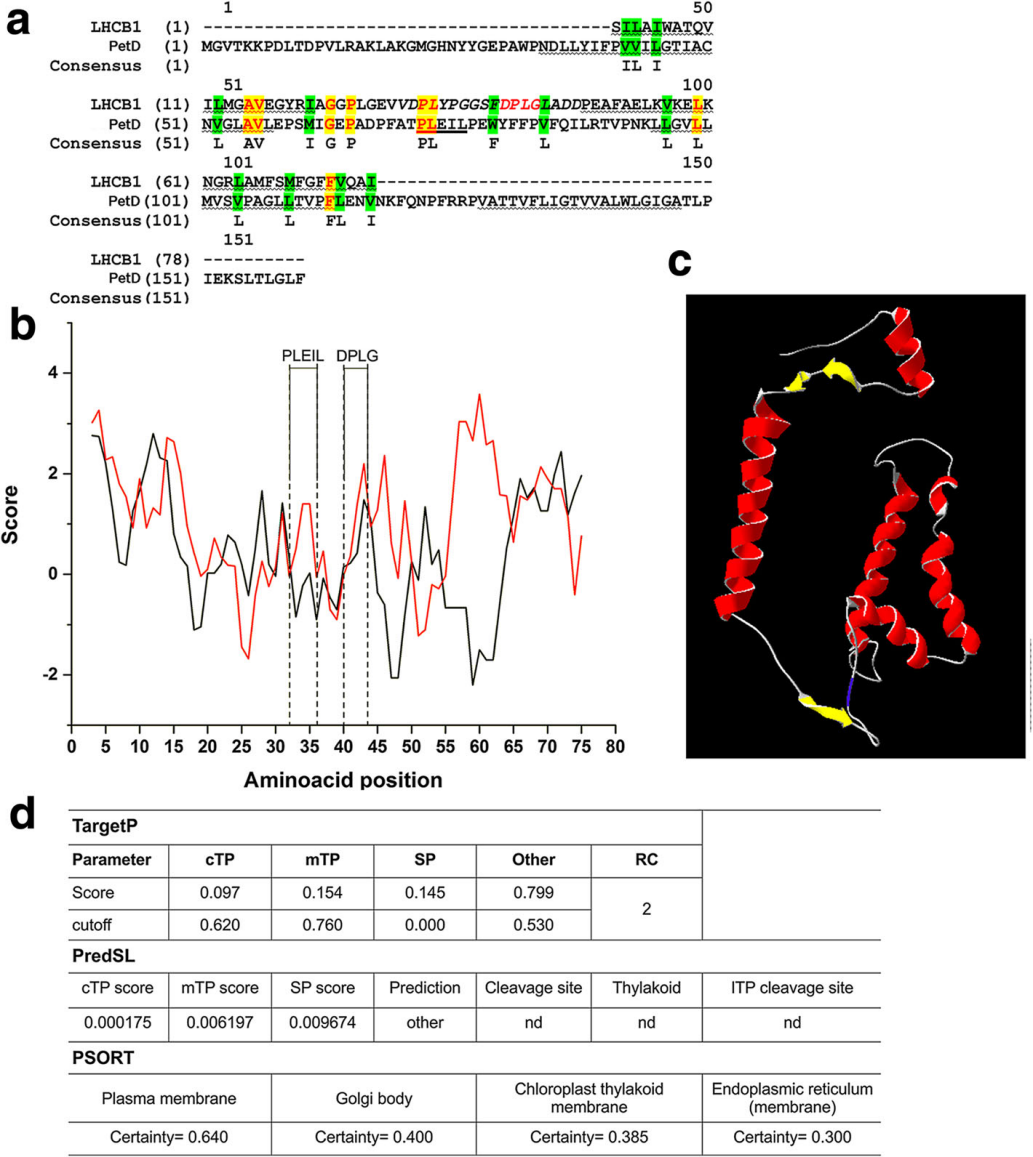
In silico analysis of potential signal sequence. **a** The PetD and LHCB1 (TM2-L18-TM3 sequence) proteins sequences from pea were aligned using MAFFT version 7 (Additional file 10: Figure S3, alignment of PetD sequences) [25]. Fully conserved residues are highlighted with a yellow background and functionally conserved residues are shown with a green background. The region corresponding to the PLEIL sequence is bold underlined, and DPLG sequence marked in red italics, TM helices are grey underlined, L18 sequence shown in capital italics [25]. **b** The Kyte–Doolittle hydropathy profile of the pea PetD (from amino acid numbers 40 to 161, (black line)) and the LHCB1, TM2-L18-TM3 sequences (red line), and the resulting Kyte–Doolittle data were aligned relative to the TM2-L18-TM3 sequence of LHCB1. Both curves display a positive score in the h-region that corresponds to relative hydrophobicity. A relative sequence numbering is given, with position 0 representing the first residue of the LHCB1 TM2, and the window size was set to 5 residues for sequences comparison. **c** The structure of PetD from *Chlamydomonas reinhardtii* (pdb 1Q90) with the PLEIL sequence is marked in blue [20]. **d** The theoretical calculation of the N-terminus of PetD protein was calculated using the bioinformatic tools (TargetP, PSORT and PredSL). Reliability class (RC) indicates the strongest prediction was found with TargetP. ND, not detected; cTP, chloroplast transit peptide

During co-translational incorporation into membranes, polypeptides that are being synthesized on the membrane bound ribosomes (nascent polypeptides) are translocated or integrated by the translocase while the ribosomes remain bound to the translocation apparatus. The membrane integration of nascent polypeptides requires a cleavable signal sequence or a TMH that provides signal anchor [14]. Post-translational incorporation takes place on cytosolic free ribosomes. Following their synthesis proteins are discharged into the cytosol, and some of these proteins last in quasi-soluble form. Whereas others containing an N-terminal hydrophobic signal sequence are identified by the signal recognition particle (SRP), and this facilitates their association with the receptor proteins and then their delivery to the pore-forming membrane protein translocation channel, where the proteins are directly integrated into the membrane. However, as in case of Light-Harvesting Chlorophyll a/b Binding Proteins (LHCPs) insertion into membrane, the post-translational SRP-dependent pathway may use an integral signal sequence as well [15]. Normally, the conserved universal SRP pathway usually mediates both co-translation and post-translational targeting. However, a unique chloroplast SRP has also been discovered in green plants [16]. This novel chloroplast pathway involves cpSRP54 and its membrane receptor cpFtsY, two GTPases that are similar to the cytosolic SRP54 and SR GTPases, and a unique 43-kDa protein, cpSRP43 [16–18]. Furthermore, a membrane-bound homologue of bacterial YidC, termed ALB3, is also involved in the SRP pathway [19]. Importantly, the mechanism and signal for this integration within the thylakoid membrane of the chloroplast-encoded cytochrome *b*_*6*_ *f* subunit IV (PetD) that consists of three transmembrane helices (TMH) (Fig. 1c) [20] remains unknown [21].

Previous analysis of thylakoid-bound ribosomes proposed a post-translational mechanism of PetD integration [12, 22]. The post-translationally integrating thylakoid proteins included the multi-spanning proteins PetD, AtpH, PsbK, and PsbZ that were proposed to integrate into membrane without the aid of a proteinaceous machinery [12]. To date, no one has verified this mechanism of PetD integration and assembly into the thylakoid membrane. Therefore, the goal of the present study was to investigate the molecular mechanism of PetD integration into the membrane in the chloroplast environment.

Our comparative analysis revealed that although PetD interacts directly with the thylakoid membrane by hydrophobic interactions, it can easily be removed with chaotropic agents. These results exclude the spontaneous pathway for insertion of PetD into the thylakoid membrane, and indicate that other proteins mediate the proper integration of PetD post-translationally. We determined that PetD is inserted into thylakoid membrane by the coordinated action of cpFTSY, cpSRP54 and ALB3 insertase. Hence, we propose that the chloroplast SRP pathway is the mechanism responsible for the post-translational membrane assembly of the chloroplast-encoded cytochrome *b*_*6*_ *f* subunit IV (PetD).

## Methods

### Bioinformatic tools

TargetP 1.1 server (www.cbs.dtu.dk/services/TargetP) was used to predict the presence of the chloroplast transit peptides (cTP) in the protein sequences and the location of potential cTP cleavage sites. TargetP has a substantially greater ability to discriminate between signal peptides and uncleaved signal anchors [23].

Kyte–Doolittle hydropathy plots [24] were generated using an online tool available at the ExPASy molecular biology server (https://web.expasy.org/protscale/). For *in-silico* multiple sequence alignments, the MAFFT version 7-alignment software (https://mafft.cbrc.jp/alignment/server/) was used. [25]. PredSL (http://aias.biol.uoa.gr/PredSL/) was used to predict a protein’s localization [26]. The PSORT Prediction tool (http://psort1.hgc.jp/form.html) that uses the overall amino acid composition, the N-terminal targeting sequence information, and the motifs, was used as a hybrid approach. This latter software uses a set of knowledge-based “if-then” rules [27].

### Isolation of thylakoid membranes and stroma fractions from intact chloroplasts

Pea seeds (*Pisum sativum*, cv Calvedon) were grown hydroponically and intact chloroplast from leaves was isolated as described previously [28]. Washed thylakoids and stromal extract was prepared from isolated chloroplasts as described previously [29]. Total chlorophyll (CHL) content was measured as described previously [30]. Prior to the experiments, reconstituted lysates were prepared by suspending the thylakoid membranes in the freshly prepared stroma.

### Preparation and analysis of free and bound ribosomes

Free ribosomes were prepared as described [22] and thylakoid-bound ribosomes were detached as described previously [31].

### Cell free transcription-translation insertion assays

The recombinant plasmid pET25b-SUIV containing the gene *PetD* was used as a template for the creation of plasmid pT7CFE1-SUIV **(**Additional file 1: Figure S1**)**. The pT7CFE1-CHis based expression construct, pT7CFE1-SUIV, was used to express the *PetD* gene. *PetD* was cloned between the MscI and BamHI restriction sites of the PT7CFE1-CHis vector (Thermo Fisher Scientific). To eliminate the need for affinity tags (His tag), a double stop codon was added at the 3′ end of the *PetD* sequence. The pT7CFE1-SUIV expression vector utilizes the T7 viral promoter and an encephalomyocarditis virus (EMCV) internal ribosome entry site (IRES) that is critical for high levels of cap-independent protein expression. Moreover, this expression vector possesses a T7 RNA polymerase promoter that functions with the T7 RNA polymerase that is included in the transcription reaction mixture. The obtained pT7CFE1-SUIV construct was confirmed to be correct by sequence analysis. The plasmid pT7CFE1-*b*_6_ is described in [11].

Coupled transcription/translation reactions of the pea *PetD* were performed as described in [11] with the following modifications: the *PetD* gene was firstly transcribed using pT7CFE1-SUIV construct as a template and the 2.0 μg of DNA-purified mRNA was used in the translation process. *PetD* mRNA was then translated in vitro in the presence or absence of thylakoid membranes, with or without the freshly prepared stromal fraction for 60 min at 30 °C in the presence of 10 μCi [^35^S]-methionine (Hartmann Analytic, Germany). Following the translation reaction, another 2.0 μg of the DNA-purified mRNA and 1 μL of manufacture “energy mix” were added for each reaction and incubated for another 30 min in order to increase the protein yield. The translation in vitro assays that were required for the post-translational insertion of PetD were performed accordingly to the method of Houben, et al. [32] with the following modifications: after 60 min of translation in the presence of 10 μCi [^35^S]-methionine, puromycin and lincomycin were added to a final concentration of 5 mM and 15 mM, respectively, and the samples were incubated for another 30 min at 30 °C. Subsequently, membranes and protease inhibitors were added, and the samples were kept for 30 min at 30 °C. The membranes were separated from the translation mix and then purified as described previously [11]. Finally, in order to remove the endogenous RNA as well as the external domains of the endogenous proteins the translation reactions, the thylakoid membranes or stroma were pre-incubated as described in [11].

### Assessment of integration of PetD into the thylakoid membranes

Assay mixtures containing 45 μL thylakoid suspension (250 μg CHL /mL) in HM buffer and 25 μL of translation mixture were incubated for 20 min at 25 °C. Following double washing of the thylakoids suspensions, the samples were analyzed. Where appropriate, incubations were pretreated with 1 unit of apyrase or sodium azide (10 mM) or nigericin (2 μM) plus KCl (10 mM).

To monitor the integration of the targeted proteins, membranes (200 μg CHL /mL) were resuspended in 2 M NaBr or 2 M NaSCN in 50 mM HEPES-KOH pH 7.7 buffer containing a protease inhibitor cocktail [33] and incubated on ice for 30 min. Next, the membranes were washed and collected (20,000×*g* for 10 min). Although the resulting membranes were solubilized in SDS sample buffer directly (pellet fraction), the supernatant fraction was precipitated in 80% acetone and then analysed by SDS–PAGE. The proteolytic assessment of protein import into the thylakoid membranes was performed as described in [34] with modifications made by Kroliczewski, et al. [11].

### Integration of the single span subunit W of PS II (PsbW)

In order to validate PetD insertion assays, we followed the thylakoid membrane integration of PsbW using the methods described in [11]. PsbW protein inserts into the thylakoid membrane by the spontaneous pathway [11].

### Crosslinking and co-immunoprecipitation of the ribosomal fractions

The ribosome-nascent chain complexes (RNCs) were cross-linked on ice using the homobifunctional *N*-hydroxysuccinade ester bis[2-(succinimidyloxycarbonyloxy) ethyl] sulfone (BSOCOES, Pierce), a membrane-permeable cleavable crosslinker or the heterobifunctional cleavable (by DTT) crosslinker N-succinimidyl-3-[2-pyridyldithio]propionate (SPDP, Pierce) by the method of Kroliczewski, et al. [11] followed by co*-*immunoprecipitation reactions using an antibody (Ab) against ALB3 or cpSRP54 proteins [11, 28]. To perform the immunoprecipitation reaction, we used 2 μg of antibody. The immunoprecipitated proteins were analysed by SDS-PAGE and Western blot or subjected to autoradiography using X-ray cassettes with a sheet of X-ray film (Kodak) [35]. The gels were dried prior to being placed on X-ray film. Furthermore, the detected bands were excised from a polyacrylamide gel and analysed by Q-TOF MS/MS spectrometry (MS) with peptide mass fingerprints (PMF), (see: Mass spectroscopy analysis section).

### PetD and PetB co-expression in cell-free assays

The cell-free expression assays were performed for 60 min to ensure synchronised expression of PetD and PetB [11] in the presence of stroma and membrane fractions followed by crosslinking with 25 mM BSOCOES or 40 mM SPDP. BSOCOES was added to the protein samples to achieve a concentration equal to 10–50 times the molar concentration of the proteins in solution. The reaction mixtures were incubated on ice for 2 h and then in Quenching Solution (1 M Tris∙HCl, pH 7.5) to a final concentration of 20–50 mM. The samples were then incubated for 15 min to quench non-reacted crosslinker and byproducts. SPDP was added to the protein samples to achieve a 4-fold molar excess of SPDP and was incubated for 60 min at room temperature. Excess of non-reacted crosslinker and reaction byproducts were removed by extensive dialysis.

The isolated membranes were then solubilized in HMS buffer containing 1% of DDM. Immunoprecipitation was performed with an antibody against PetB as described in [28]. Afterwards, base-cleavage of BSOCOES was performed by increasing the pH of the solution containing the BSOCOES conjugate to 11.6 with NaOH, which was incubated for 2 h at 37 °C, but cross-links created using SPDP reagents can be cleaved with 25 mM DTT at pH 4.5 Obtained samples were dialyzed into HMS buffer for subsequent analysis. The proteins were then identified with MS.

### Preparation of stroma fractions without the cpSRP54 protein

Protein A Sepharose CL-4B was prepared according to the standard protocol of GE Healthcare. An antibody against cpSRP54 (5 μg mL^−1^) was incubated 30 min at room temperature with 2 ml of isolated stroma fraction. Subsequently, 20 μL of protein A-Sepharose CL-4B resin in 20 mM sodium phosphate buffer, pH 7.0 was added and the incubation was continued with very mild rotation at 4 °C for an additional 90 min. The resin was collected by centrifugation at 500×g, 5 min, 4 °C and the supernatant fraction containing stromal proteins without cpSRP54 was collected and immediately used in a PetD insertion experiment.

### Mass spectroscopy analysis

For identification, the proteins were analyzed as described in [11, 28]. Tandem mass spectrometry experiments were carried out on a Q-Tof Premier mass spectrometer in conjunction with a nanoACQUITY UPLC system (Waters Corp) to obtain peptide information. Tandem mass spectrometry output lists of precursor and product ion information were used for NCBI database searching using the program Mascot Distiller (version 2.1, Matrix Science) [36].

In order to identify inserted proteins using mass spectroscopy following the insertion assays, the proteins were analyzed by SDS/PAGE and autoradiography. A gel band containing the radioactive polypeptide was excised. Moreover, at the same time, positive and negative control samples for mass spectroscopy analysis were prepared. As positive control, proteins from the molecular weight marker were used, while a piece of empty gel with no protein present was used as a negative control in order to determine any contamination that may have occurred during sample processing. Proteins were first extracted from polyacrylamide gels using electroelution (D-Tube, Novagene). Following electroelution, salts, SDS, and dye were removed by dialysis in D-Tube and the resulting proteins samples were then concentrated using an Amicon Ultra spin column.

All samples were analyzed by MALDI-TOF using the 4800 Plus MALDI-TOF/TOF™ Analyzer (Applied Biosystems) as well. Mass spectra for each gel slice were obtained in three replicates. Additionally, the proteins were digested by trypsin and analyzed by MS with PMF. The analysis procedure included washing, destaining, reduction and alkylation as described in [37].

### Protein analyses

Protein concentrations were determined with the BCA™ protein assay kit (Pierce). Following the normalization of protein concentrations, proteins were separated via Tricine/Tris SDS-PAGE [11, 38] and transferred to nitrocellulose membranes using a semidry blotting system [11].

For protein detection, the following antibodies were used: polyclonal antibodies against PetB (antibodies raised against the 10 C-terminal amino acids, IRKQGIFGPL, of pea PetB) [28], cpSecY (antibodies raised against the 21 C-terminal residues of CRAEIISQKYKNIELYDFDKY of pea cpSecY) [39], ALB3 (antibodies raised against the 50 amino acid, PLTKQQVESTLAMQNPQPKIKAIQERYAGNQERIQLETSRLYTQAGVNPL, of the stromal protein sequence located between the first and second transmembrane (TM) domains) [40]. Antibodies were prepared in rabbits injected with the appropriate synthetic peptide (GenScript). The overexpressed and purified cpSRP54 [28] and a 21-amino acid synthetic peptide (YPIFAQQGYENPREATGRIVCANC), corresponding to the highly conserved N-terminus of the mature pea PetA [41], were used to prepare chicken (IgY) anti-cpSRP54 and anti-PetA antibodies, respectively. Antiserum was purified using the Pierce™ Chicken IgY Purification Kit (Thermo Scientific) followed by affinity-purification method against the antigen (Thermo Scientific). IgY antibodies was labelled with horseradish peroxidase using EZ-Link™ Plus Activated Peroxidase Kit (Thermo Scientific).

For antibody preparation, the antigen affinity purification (GenScript) or AminoLink™ Immobilization Kit (Thermo Scientific) were used. Affinity purification permits isolation of antibodies against the immunizing antigen and thereby eliminates cross-reactivity (‘unintentional’ binding) that would be present in the unpurified serum. The results of cross-reactivity studies are shown in Additional file 2: Figure S2. Antibodies were evaluated based on yield after affinity purification and the analysis of the specific activity of the purified antibodies. The titers of antigen-specific antibodies were determined by ELISA in 96-well Immulon 2HB flat bottom microtiter plates using the immunogen (Thermo Scientific).

The PetD protein presence in membrane and in immunoprecipitated complexes was confirmed by autoradiography followed by MS analysis with PMF since the antibodies directed against the PetD protein are unavailable [11]. Prior to autoradiography, gels were stained with Coomassie brilliant blue to confirm equal protein loading and then dried under vacuum. Importantly, we always used the same exposure time so that the image intensity was used as a consistent guide to the exposure required for subsequent autoradiography. Molecular masses were determined by comparison to molecular weight ladder (MW). For autoradiography, the MW bands were gently marked on the X-ray film (Kodak) with a pencil and the X-ray film was placed over the dry gel and exposed for 24 h. Quantitative densitometry of Western blot and autoradiography were performed using Image Lab software v. 4.1 (BioRad). The antibody bound proteins were detected by a chemiluminescence reaction using ECL (enhanced chemiluminescence, Amresco).

## Results

### Bioinformatic analysis of PetD did not identify a membrane targeting signal sequence

Using TargetP tools, PetD was predicted to be a protein without a specific signal sequence or anchor sequence (Fig. 1d). Additionally, we used PredSL and PSORT software tools which combine multiple methods in order to predict a protein’s localization to the chloroplast and the thylakoids as well as to the mitochondrion or the secretory pathway [26]. Unfortunately, neither of the predictive methods used could identify the presence of a signal sequence potentially responsible for PetD transfer and its insertion into the thylakoid membrane (Fig. 1d).

The N-terminal regions of membrane proteins often serve as a membrane anchor, whereas the majority of known membrane proteins are translocated into membranes by a cleavable or non-cleavable signal sequence. In order to detect a potential non-cleavable signal within the PetD primary structure, the Kyte–Doolittle analysis was performed with a sequence window size of 5 amino acids that is appropriate for detecting a potential hydrophobic signal at N-terminus. The resulting data were aligned relative to the TM2-L18-TM3 amino acid sequence of the light-harvesting chlorophyll a/b binding proteins subunit B (LHCB1) (Fig. 1a). During the post-translational cpSRP-dependent insertion of LHCP, LHCP affiliates with cpSRP54 and cpSRP43 to form a stromal ‘transit complex’. Formation of this complex is governing by a sequence of specific recognition and interaction events. The interaction between LHCP and cpSRP43 involves a conserved 18 amino acid span, termed L18, that is located between two TMH, (TM2 and TM3) of LHCP. Hence, the L18 provides an internal targeting signal for a highly specific interaction between LHCPs and cpSRP43. After transit complex assembly, a third protein, cpFtsY, is assumed to target the transit complex to the thylakoid membrane [8].

The results of the Kyte–Doolittle analysis indicate that PetD does not contain a non-cleavable signal sequence. However, as shown in Fig. 1b, the average hydropathy plots of the compared PetD and LHCB1 sequences show similar profiles within the first 40 amino acids, but at a different hydrophobicity level. A consensus amino acid sequence, PLEIL (ankyrin repeats motive), is present in both proteins. Figure 1c shows the topography of the PLEIL sequence, marked in blue in the PetD structure [20]. However, the PetD sequence is missing DPLG motif that is responsible for cpSRP43 binding by LHCB1 [42]. Moreover, the average hydropathicity (GRAVY) values of these PLEIL sequences were similar (0.817 and 0.989 respectively).

For the most of the outer envelope proteins, their targeting plastid compartments (inner envelope membrane, stroma, and thylakoid) depend on the presence of a cleavable transit sequence in the precursor N-terminal region. Furthermore, the reports of Miras, et al. [43] suggest that neither the N-terminal nor the C-terminal sequences are essential for chloroplastic membrane localization of the ceQORH (Chloroplast Envelope Quinone Oxidoreductase Homologue) that has non-canonical sequence for chloroplast membrane integration. Therefore, we compared the PetD sequence with the non-canonical non-cleavable transit peptides of ceQORH. These analyses did not identify any sequence similarities between PetD and ceQORH within the range of 75 N-terminal amino acids. However, the Kyte–Doolittle hydropathy profiles of the first 75 amino acid of PetD and ceQORH protein (Additional file 3: Figure S4A) show some similarities between first 35 amino acid region of the PetD amino acid sequence is shifted by 8 amino acids toward the C-terminal end (Additional file 3: Figure S4B).

### PetD is not inserted directly into the membrane in an unassisted posttranslational manner

**Fig. 2:**
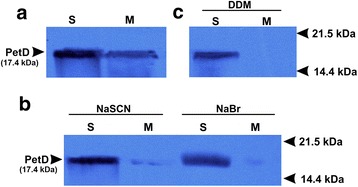
Autoradiograph of thylakoid membrane fractions after integration of radiolabeled PetD. **a** PetD in the presence of thylakoid membrane supernatant (S) and membrane pellet (M) after fractionation. **b** The supernatant (S) and membrane pellet (M) fractions after insertion of PetD and chaotropic agent (NaSCN or NaBr) treatment. **c** The supernatant (S) and membrane pellet (M) fractions, respectively, after insertion of PetD, but in this case, the translation mixture was solubilised in the presence of 0.2% DDM before insertion into membrane. All experiments were repeated twice and each sample contained 15 μg of total protein that was loaded per well. The MW of radiolabeled product was determined by MALDI-TOF

To verify that the PetD protein integration into the pea thylakoid membrane can occur by the spontaneous pathway, the protein was expressed in a cell-free system as described in [11] in the presence of proteinase K-treated thylakoid membranes, but in the absence of the stromal fraction. During this translation protocol, 30% of the synthesized PetD protein was associated with the thylakoid membrane fraction (Fig. 2a). The molecular weight of inserted and integrated PetD protein was determined by mass spectrometry (Additional file 4: Figure S5A). MS analysis confirmed that the PetD construct is correctly expressed in the in vitro translation system. The molecular weight observed for PetD (17,487 Da) in MS analysis agrees well with the theoretical molecular weight of the monomeric species (17,476 Da).

The PetD protein integration with thylakoid membrane was further tested with use of strong chaotropic salts like NaBr and NaSCN that remove proteins peripherally bound to the membrane without membrane disassembly [32, 44, 45]. As shown in Fig. 2b, the membrane associated PetD was almost completely removed. Moreover, in order to facilitate spontaneous membrane integration, the synthesized PetD protein was solubilised in the presence of 0.2% DDM, and then mixed with purified thylakoid membrane. This approach, however, did not increase the resistance of the membrane-associated PetD to chaotropic removal (Fig. 2c). As a control for spontaneous protein insertion, we tested whether PsbW of the photosystem II or PetB can be spontaneously inserted into the thylakoid membrane [11, 46]. Both control experiments were performed under the same conditions as those for the petD protein. These experiments confirmed the previous observation that PsbW is indeed inserted into the thylakoid membranes by the spontaneous pathway. Furthermore, we also confirmed that PetB cannot be spontaneously inserted into thylakoid membranes (Additional file 5: Figure S6). All above results indicate that the native PetD cannot incorporate into thylakoid membranes spontaneously.

### Post-translation import of PetD into the thylakoid membrane is SRP-dependent

The previous comparative analysis of ribosomal fractions bound to thylakoid membranes and from stroma indicate that PetD is translated on free ribosomes exclusively [22], suggesting a post-translational mechanism for protein membrane integration [12, 22]. Therefore, since the results of the thylakoid import assays excluded the spontaneous pathway for PetD membrane integration, we next verified the proposed post-translational translocation of PetD using a cell free in vitro system.

**Fig. 3:**
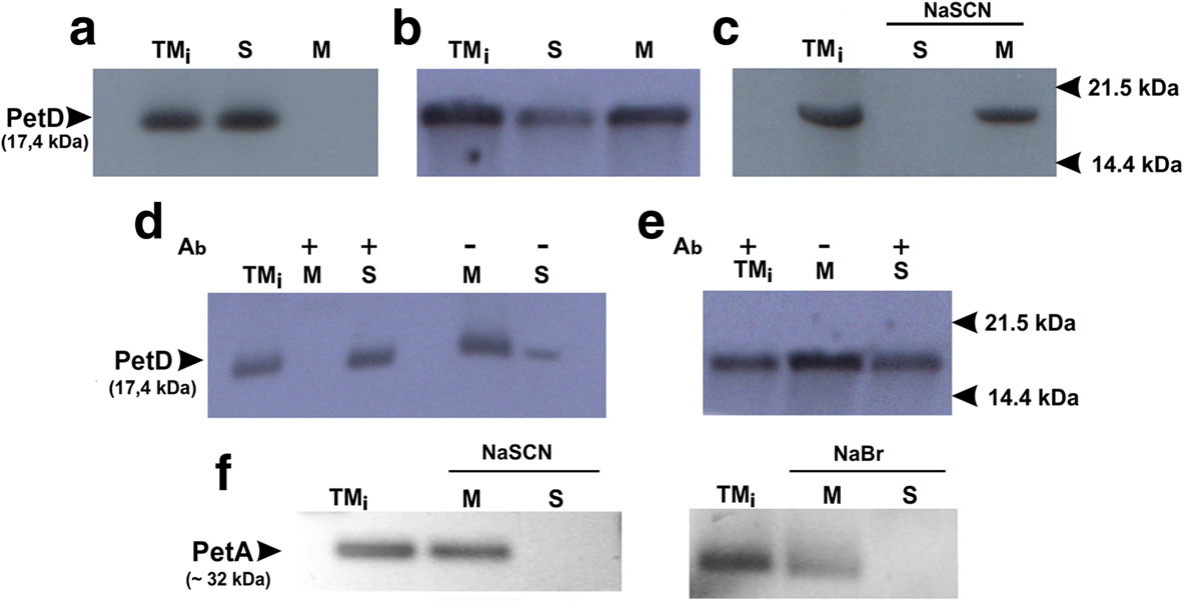
Import of the PetD protein into isolated pea thylakoid. **a** Insertion of radiolabeled PetD in the presence of thylakoid membrane, supernatant (S) and membrane pellet (M) after fractionation, the total translation mixture is marked TM_i_, **b** TM_i_- insertion of the translated PetD in the presence of thylakoid membrane and stromal protein fractions, and the S-supernatant and M-membrane pellet after fractionation. **c** Chaotropic extraction of inserted PetD by NaSCN, TM_i_-fraction after PetD insertion (control); S-supernatant and M-membrane pellet after fractionation. **d** Insertion of PetD in the presence of thylakoid membrane and Ab against ALB3 protein (denoted with “+”). TM_i_-fraction after PetD insertion (control); S-supernatant and M-membrane pellet after fractionation. **e** Insertion of translated PetD in the presence of thylakoid membrane and Ab against cpSECY protein (denoted with “+”). TM_i_-fraction after PetD insertion (control); M -membrane pellet; S-supernatant after fractionation. **f** Western blot analysis of chaotropic extraction of PetA subunit from thylakoids membrane with inserted PetD. TM_i_-fraction after PetD insertion (control); S-supernatant and M-membrane pellet after fractionation. NaSCN or NaBr was used as the chaotropic agent [33]. The experiments were repeated at least twice. Each lane was loaded with 15 μg of total protein aliquot. Membranes were incubated with appropriate Ab before insertion started and washed twice. Fig’s **a − e** present autoradiograph analysis. MW of radiolabeled product was determined by MALDI-TOF

PetD expression was performed in a cell-free system by using the transcription–translation procedure where transcription and translation reaction were separated in space and time (see Materials and Methods, section: The cell free transcription-translation insertion assay). Moreover, during the post-translational insertion experiments, translations were performed in the absence of thylakoid membranes. As shown in Fig. 3a, without the stroma fraction the translation product (PetD) did not integrate into the membranes and was detected in the supernatant only. However, when the stroma fraction was present together with the membranes, the PetD was localized in the membrane fraction (Fig. 3b). Importantly, both inserted PetD and PetA (control) are resistant against all extraction procedures (Fig. 3c and f) [33]. Moreover, this result clearly shows that proper PetD membrane incorporation occurs via the post-translational mechanism and requires the stroma factors.

Hence using specific antibodies, we examined the mechanisms responsible for the membrane integration of PetD. Pretreatment of the thylakoids membrane with Ab against ALB3 prevented insertion of PetD into the membranes (Fig. 3d), suggesting that ALB3 is required for this process. However, although a significant level of PetD insertion was achieved in the presence of cpSecY Ab (Fig. 3e), it was still lower than in the control (Fig. 3e). Hence, the Ab against cpSecY only partially prevents cpSecA-dependent translocation of PetD into the membrane by the Sec pathway since this antibody blocks cpSecA binding to cpSecY without functional inhibition of cpSecY [39, 47, 48].

**Fig. 4:**
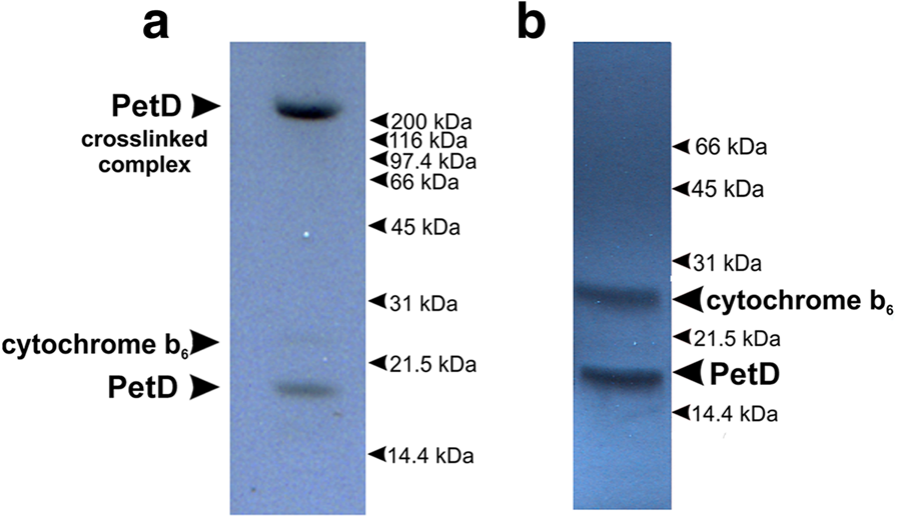
Analysis of PetD and cytochrome *b*_*6*_ co-insertion into the thylakoid membrane. **a** Autoradiograph of thylakoid membrane fractions are shown after insertion of radiolabeled PetD and cytochrome *b*_*6*_ co-expressed in cell-free assay. **b** Analysis of isolated high molecular weight PetD crosslinking complex is shown after immunoprecipitation with antibody against cytochrome *b*_*6*_ and cleavage of crosslinker (BSOCOES). The interacting proteins were identified using MS with Mascot Distiller analysis. These proteins were confirmed as PetD and PetB. Moreover, the analysis resulted in the identification of a number of peptides that were impossible to assign to a specific protein, as well as proteins with very low total score [36]. The molecular weight of the autoradiography band was determined by MALDI-TOF. For size detection, the molecular weight ladder bands were marked on the X-ray films with a pencil

The current model of cytochrome *b*_*6*_ *f* complex assembly assumes the partial transcriptional activation of psbBNH-*petBD* operon within the *PetB and PetD* genes [49]. Following transcription, the *PetB* and *PetD* mRNA are translated into the polypeptides. As a result of this process, both proteins are incorporated into the membrane and form the polytopic monomeric core of the cytochrome *b*_*6*_ *f* complex. Hence, in order to validate the proposed mechanism for formation of PetD-PetB core complex, both genes were transcribed/translated in the cell-free system in the presence of the stroma and thylakoid membranes fractions (see PetD and PetB co-expression in cell-free assay in Materials and Methods section). After 1 h of translation, a membrane permeable and cleavable crosslinker (BSOCOES) were added. As shown in Fig. 4, the cotranslation of *PetB* and *PetD* resulted in detection of the PetD translation product with higher molecular weight than compared to PetD translated alone (Fig. 4a). Furthermore, the immunoprecipitation with an Ab against PetB of cross-linked isolated membrane complexes, followed by MALDI-TOF mass analysis confirmed that the radiolabeled complex contains a PetD-PetB complex (Fig. 4b and Additional file 4: Figure S5B).

**Fig. 5:**
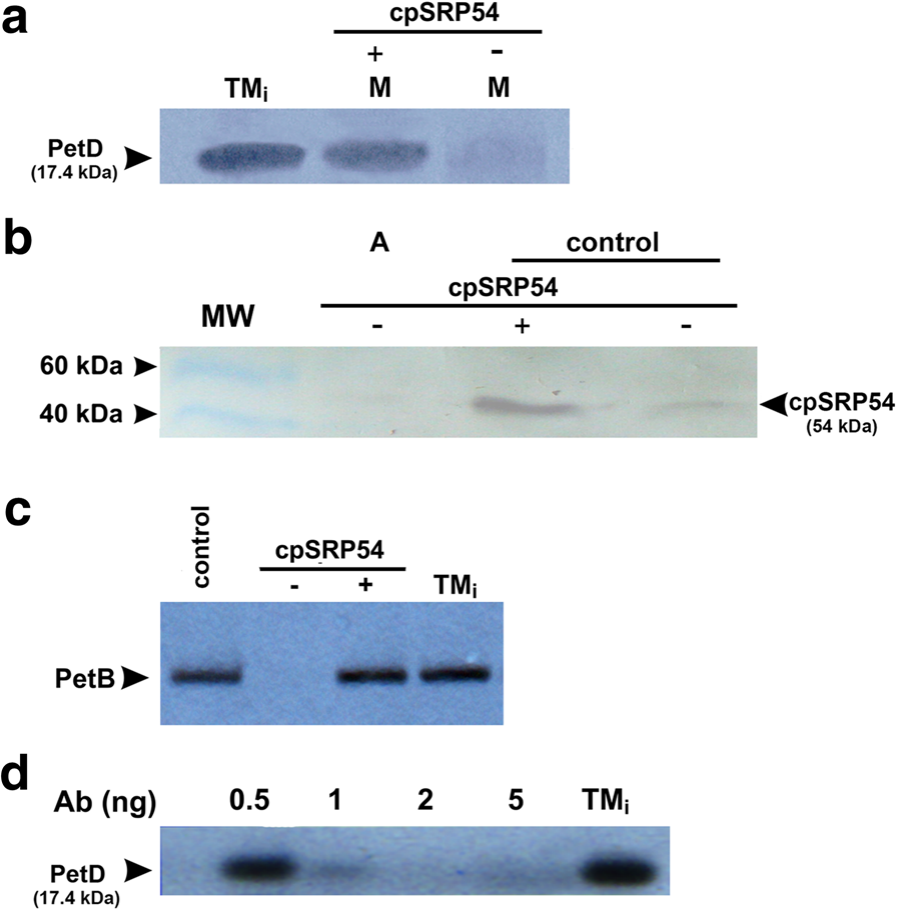
PetD insertion into the thylakoid membrane after removal of the cpSRP54 protein. Analysis of PetD insertion in the presence of thylakoid membrane and stroma fraction after the cpSRP54 protein (denoted with “-”) was removed by immunoprecipitation. **a** Autoradiograph of thylakoid membrane fractions after radiolabeled PetD was inserted in the presence of thylakoid membrane and stroma fraction (M) with or without the cpSRP54 protein; TM_i_ -total translation mixture. The MW of radiolabeled product was determined by MS. **b** Western blot analysis of the stroma fraction is shown with or without the cpSRP54 protein. MW- molecular weight marker; A- stroma fraction used in experiment and stroma fractions with or without the cpSRP54 protein (control). **c** Autoradiograph of thylakoid membrane fractions are shown after insertion of radiolabeled PetB according to the method described in [11]. PetB insertion in the presence of thylakoid membrane and stroma fraction with (control); without the cpSRP54 protein and after cpSRP54 recovery to stroma fraction; total translation mixture (TM_i_) was used as a control; **d** Autoradiograph of thylakoid membrane fractions are shown after insertion of radiolabeled PetD in the presence of thylakoid membrane and stroma fractions. Prior to the experiment, the stroma protein fraction was incubated with different concentrations of antibody against cpSRP54 for 4 h at room temperature with gentle mixing. The total translation mixture (TM_i_) without antibody was used as a control

As shown in Fig. 5, when the posttranslational insertion of PetD was performed in the presence of thylakoid membrane and stroma fractions devoid of the cpSRP54 protein, the PetD protein did not incorporate into membranes. Although a very weak signal is observed (Fig. 5a), it probably results from the incomplete removal of the cpSRP54 from stroma (as shown in Fig. 5b). Furthermore, two control experiments were set up to ensure the integrity of the method. In the first experiment, shown in Fig. 5c, we tested the co-translational insertion of PetB in the presence or absence of the cpSRP54 protein. As expected, we did not observe PetB insertion in the absence of the cpSRP54 protein. The goal of the second experiment (Fig. 5d) was to determine the impact of using a different concentration of antibody against cpSrp54 during PetD posttranslational insertion. We found that with increasing concentrations of antibody, there was corresponding decrease in the level of PetD embedded in the thylakoid membrane.

Furthermore, to identify the chloroplast proteins that governed PetD import, a chemical cross-linking analysis followed by immunoprecipitation using antibodies directed against ALB3 or cpSEC54 was performed followed by MS detection. The results of immunoprecipitation analysis combined with MS-PMF and Mascot Distiller analysis are shown in Table 1. The results show the association of cpSRP, cpFtsY and ALB3 proteins with PetD during its insertion into the membrane. Furthermore, the analysis resulted in the identification of several hundred of peptides that could not be assigned to specific proteins or to proteins with low score (Additional file 6: Table S1).

**Table 1:** The selected proteins crosslinked to PetD identified by peptide mass fingerprinting

No.	Protein^a^	Protein score^c^	Peptide identified by MS^b^	Annotation in database
1	PetD	750	MGVTKKPDLTDPVLR LLGVLLMVSVPAGLLTVPFLENVNK SLTLGLF	AAD41889
2	cpSRP54	517	LDGDSRGGAALSVK EVSGKPIKLVGR TEQQVSQLVAQLFQMR	AAC64109
3	cpSRP43	95	KADEQALSQLLEDR LLAEAGADLDHRDMR	O22265
4	ALB3	473	ALQQRYAGNQER YAGNQER	NP_001189626
5	cpFTSY	398	DALKESVLEMLAK KPAVIMIVGVNGGGK TGCEIVVAEGDK LHTNYSLMEELIACK	NP_566056
6	^c^PetB	480	LEIQAIADDITSK VYLTGGFK IVTGVPDAIPVIGSSVVELLR	AIK21467

^a^Chloroplast proteins which produced the highest scores are shown

^b^Peptide identifications were accepted if they could be established at greater than 80.0% probability

^c^Protein shown in double expression experiments. Analyses were repeated at least twice. MASCOT search and protein identification criteria were previously published in [11, 28, 36]

**Fig. 6:**
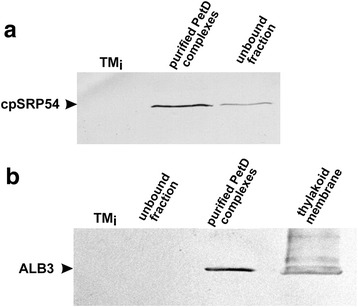
Immunoblotting analysis of the cross-linked PetD complexes after affinity purification. **a** The fractions were immunoprecipitated with antiserum directed against the ALB3 and then blotted for cpSRP54. the total translation mixture (TM_i_) was used as a control; **b** The fractions were immunoprecipitated with antiserum directed against the cpSRP54 and then blotted for ALB3. Total translation mixture (TM_i_) was used as a control. Both experiments were twice repeated. An aliquot of 15 μg of total protein was loaded on each lane. Antibodies were immobilized to Protein A Sepharose beads

These MS results were further confirmed by Western blot analyses of the cross-linked complexes followed by immunochemistry. As presented in Fig. 6a, the Ab against cpSRP54 detected large amounts of cpSRP54 in the isolated membrane fractions. A smaller, but significant pool of cpSRP54 was detected in the immobilized ALB3 antibody unbound fraction as well. Importantly, the immunopurification of the cross-linked PetD complexes with antiserum directed against the cpSRP54 (Fig. 6b) allowed for the detection of ALB in the purified PetD complexes.

The proteins we identified by MS were previously recognized as crucial for SRP-mediated post-translational transport of LHC proteins into the thylakoid membrane [16]. Hence, our results of MS (Table 1) and autoradiograph analyses (Fig. 3) are strong indicators that PetD import into the membrane occurs through the post-translational SRP pathway.

**Fig. 7:**
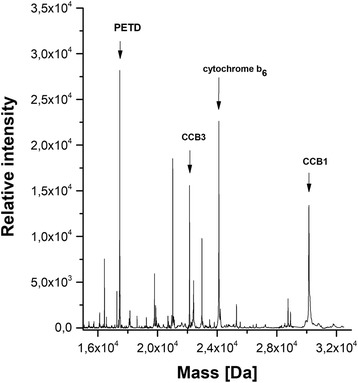
MALDI-TOF mass spectra. Mass spectrum was obtained after isolation of PetD and PetB crosslinked (SPDP) complexes from thylakoid membranes followed by immunoprecipitation and cleavage with 25 mM DDT solution [12]. The unconjugated proteins solution was analysed by MALDI-TOF. The intensity (peak height on the y-axis) of the resulting MALDI spectrum is not quantitative

MALDI-TOF mass analysis (Fig. 7) of intact proteins crosslinked by SPDP, immunoprecipitated and then cleaved with 25 mM DTT from the petD complexes shows two of CBB proteins (CCB1 and CCB3) that play important roles in guidance of apocytochromes and in haem groups for their covalent linkage by the cytochrome-c-hem lyase [6]. Importantly this analysis confirmed that after insertion PetD and PetB form a complex at the membrane with predicted size of 38.67 kDa (Fig. 4a, Table 2 and Additional file 4: Figure S5B). This result was confirmed by MS analysis of a tryptic digested PetD complex (Table 2).

**Table 2:** Analysis of proteins co-immunoprecipitated with PETD-cytochrome *b*_*6*_ complexes after insertion into thylakoid membranes

No.	Protein^a^	Protein Score^b^	Peptide identified by MS^c^	Annotation in database
1	PetB	480	LEIQAIADDITSK VYLTGGFK IVTGVPDAIPVIGSSVVELLR	AIK21467
2	PetD	628	KPDLTDPVLRAK LLGVLLMVSVPAGLLTVPFLENVNK FQNPFR RPVATTVFLIGTVVALWLGIGATLPIEK	AAD41889
3	CCB1	225	IVNKTFVK LLDEVGNKAPNQVAGEVLSFFTR EDGTLSEIVVQGDDQQVEQMRK	Pisum_sativum_v1_Contig2650 [64]
4	CCB3	99	LMILADLDPATAK FPYVIAYAPTEPLLVPTRK	*Pisum sativum*_csfl_reftransV1_0082495 [64]
5	GUFP	43	VIASEALSAIR	Q9FNM5
6	GAPDH	117	TFAEEVNEAFR ELGIDLVIEGTGVFVDR	CAA33264

For the immunoprecipitation of complexes, antibody against cytochrome *b*_*6*_ was used. Bound proteins were directly analyzed by MS with PMF (data shown in Additional file 6: Table S2

^a^Chloroplast proteins which produced the highest scores are shown

^b^Others proteins with higher scores than 40 were not observed after cross-linking

^c^Peptide identifications were accepted if they could be established at greater than 80.0% probability. Protein shown are from the double expression experiments. Analyses were repeated at least twice. MASCOT search and protein identification criteria were previously published in [11, 28, 36]

## Discussion

In chloroplasts, there is significant intraorganellar sorting of proteins, and because of the endosymbiotic origin of chloroplasts, the process of translocating a protein from the stroma of the chloroplast to the thylakoid lumen and membrane is topologically equivalent to prokaryotic protein secretion [50, 51]. The related signals are analogous to the SP for protein secretion. The signals are organized in a bipartite presequence with the cTP at the N-terminus, followed by the SP-like signal, which is resolved only after the processing of the cTP fragment. Furthermore, the luminal transfer peptide (lTP) that is present in proteins translocated to the thylakoid lumen, is very similar to the SP signal [50].

Over the past decade, several computational methods have been developed to predict subcellular localization of plant proteins [52]. Gómez, et al. [53] have shown that the SignalP software, using Gram-negative bacteria settings (TargetP contains SignalP prediction algorithm), correctly predicts the processing site for proteins that are imported into the thylakoid membrane via the “spontaneous” pathway. This observation suggests that the mechanism of spontaneous import of thylakoid protein into membrane may be related to the mechanism of substrate secretion in Gram-negative bacteria. However, our analysis shows that TargetP and PredSL suggested “other” locations for the PetD protein. Only the PSORT software identified the chloroplast thylakoid membrane as a possible targeting location for PetD, but the score of this analysis was low. Hence, it can be concluded that none of the used programs could convincingly predict the transit peptide processing site of PetD.

Because PetD lacks a canonical signal sequence for protein translocation and membrane integration, we examined PetD primary structure for other non-canonical motifs that may govern its membrane import. The PetD sequence was compared with non-canonical non-cleavable transit peptides of ceQORH. These analyses did not identify sequence similarities between PetD and ceQORH. However, when we compared both these sequences, we observed some similarity with the ceQORH sequence in their hydrophobic and hydrophilic properties. Concluding from this analysis about signal or anchor sequences, however, would be speculative at best. Therefore, further studies will be required to understand the role of the petD N-terminal sequence during the membrane integration process. Furthermore, Miras, et al. [43] suggested that a domain of the ceQORH bacterial ancestor may have evolved so as to exclude the general requirement for an N-terminal plastid transit sequence.

The PetD primary structure is related to amino acid sequence TM2-L18-TM3 of LHCB1 that is responsible for cpSRP43 binding (Fig. 1a). The LHCB1-cpSRP43 interaction is predominantly hydrophobic and a DPLG motif between the TM2 and TM3 is known to be required for the binding with cpSRP43 [42]. Although, the hydropathy profiles of amino acids between the second and the third helix of PetD and LHCB1 are very similar, PetD lacks the DPLG consensus sequence. The lack of a DPLG motif in the PetD sequence suggests that this protein does not directly bind cpSRP43. However, the PLEIL motif may mediate protein-protein interactions as well and therefore limit the need for this other motif [54].

Because the bioinformatic analyses did not identify the PetD sequence responsible for directing this protein to the appropriate insertion pathway, we first determined if PetD can be spontaneously integrated into the thylakoid membrane. We performed a comparative analysis of PetD insertion into purified proteinase K-treated thylakoid membranes. Our criteria for determining the correct mechanism of insertion were that the protein should be integrated within the membrane and therefore resistant to chaotropic extraction. As a control, we tested if PsbW that is integrated into the thylakoid membrane by a spontaneous mechanism and PetB protein that is integrated into the thylakoid membrane in a co-translational manner are resistant to chaotropic extractions. Our results indicated that chaotropic agents easily extracted PetD from the thylakoid membrane, whereas both control proteins were resistant to chaotropic extraction. Since the native PetD protein is resistant to chaotropic agent’s extraction [33], our results exclude spontaneous insertion of PetD into the thylakoid membrane.

PetD is one of the major chloroplast-encoded subunits of cytochrome *b*_*6*_ *f* and has been proposed by previous studies to be post-translationally integrated with thylakoid membrane [12, 22]. However, this hypothesis was never verified. Therefore, the aim of this study was to identify the mechanisms responsible for PetD thylakoid membrane integration using a cell free transcription/translation system in different experimental conditions (in the presence of thylakoid membranes or thylakoid membranes and stroma proteins fraction or without thylakoid membranes) [11].

The analysis of the ribosomal fraction binding with thylakoid membranes and from stroma indicated that PetD is translated from the dicistronic *PetB–PetD* transcript on free ribosomes exclusively [22]. Also, we confirmed that PetD was not found in the membrane bound ribosomes and was predominantly synthesized by free ribosomes (Additional file 7: Figure S7). Hence, we assume the possibility of a strictly post-translational integration of PetD into the thylakoid membrane by the GTP-dependent SRP pathway. Inspection of our data demonstrate unequivocally that the post-translationally inserted PetD is resistant to chaotropic extraction like a native PetA and suggests that PetD is integrated and anchored in the membrane by strong electrostatic and hydrophobic forces.

In order to verify this hypothesis, we followed the PetD thylakoid membrane integration in vitro. Furthermore, to identify chloroplast proteins that could govern PetD import, a chemical cross-linking protocol followed by immunoprecipitation approach combined with mass spectrometry detection was applied. Using this approach, we show that two chloroplast GTPases, cpSRP54 and cpFtsY and the chloroplast translocase ALB3, are involved in the PetD membrane integration process. cpFtsY is a GTPase receptor that is partitioned between the target membrane and the soluble phase and its function in thylakoid biogenesis is to target integral membrane proteins to thylakoids. Moreover, cpFtsY plays a critical role in the intramolecular communication that regulates cpSRP receptor functions at the membrane [55]. During the posttranslational targeting the preserves hydrophobic substrates in an integration competent form. At the membrane, the substrate is identified by the translocase and consequently discharged from the cpSRP54 for integration [56]. Importantly, during the import assay, only the Ab against ALB3 [57] completely prevented insertion of PetD into the thylakoid membrane. Hence, blocking ALB3 association with cpSRP and cpFtsY and inhibiting PetD protein integration suggest that formation of a protein complex containing cpSRP, its receptor, cpFtsY, and ALB3 translocase is required for proper PetD integration [57]. However, use of antibodies against cpSecY only partially limited PetD membrane import [9, 39]. Although, these results suggest that cpSecY is involved in membrane integration of PetD, the limited inhibition of PetD integration in this case may be due to other factors not related to protein transport and incorporation of proteins into the thylakoid membrane. Moreover, immunoprecipitation of cross-linked proteins with an Ab against ALB3 or cpSEC54 combined with MS and PMF analysis performed in the presence of the stroma fraction detected cpSRP43 to be only “associated” with cpSRP54-PetD complexes. However, we do not observe this protein in the isolated membrane bound cpSRP54-PetD complexes.

The SRP complexes in the chloroplast stroma, heterodimers consisting of cpSRP43 and cpSRP54, are observed in plants only [16, 18] and responsible for LHCPs import into the thylakoid membrane [58]. Dünschede, et al. [59] suggested that the coevolution of LHCPs and cpSRP43 developed autonomously of the complex arrangement with cpSRP54, and that the interaction between cpSRP54 and cpSRP43 developed more recently during the progression from chlorophytes to land plants. Importantly, stroma also contains a free cpSRP54 fraction that is not bound with cpSRP43, and that can bind to the 70S ribosome and participate in cotranslational integration of membrane proteins [17]. Hence, our data suggest that PetD membrane integration is mediated mainly by cpSRP54.

Based on the results of the analysis by mass spectrometry, we cannot exclude that cpSRP43 is involved in this process before the cpSRP54-PetD complex formation. However, since PetD lacks the DPLG consensus, the cpSRP43 presence in the stroma fraction may be an experimental artifact as well. Although, Dünschede, et al. [59] demonstrated that the cpSRP system in *Chlamydomonas reinhardtii* differs from that of land plants in that cpSRP43 is complexed to cpSRP54, Tzvetkova-Chevolleau, et al. [60] showed that cpSRP43’s role in targeting LHCPs to the thylakoid membranes is independent of cpSRP54/cpFtsY. Hence, the possible involvement of cpSECY insertase in the PetD membrane import process will also requires further study.

Interestingly, we detected two cofactor assemblies on complex C protein subunits, CCB1 and CBB3 which are involved in PetB biogenesis, in double expression assays of isolated crosslinked PetD-PetB complexes that were analyzed by MS [61] (Additional file 8: Table S2). Additional file 9: Figures S8 and S9 show the sequence alignment of CCB1 and CCB3. After its insertion into the thylakoid membrane, apocytochrome *b*_6_ binds its two non-covalent *b*-type haems [6]. The integration of both haems to apocytochrome *b*_6_ occurs spontaneously and does not require adjuvant proteins involvement [62, 63]. However, the consequent binding of haem *c*_i_ via an untypical single thioether bond engages an enzymatic CCB system [6].

## Conclusions

**Fig. 8:**
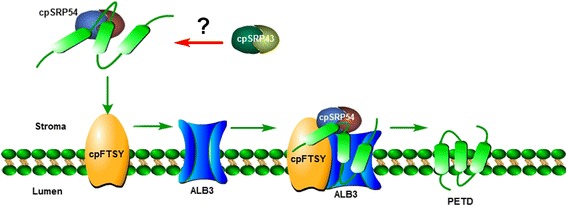
Proposed model for targeting and insertion of PetD by the post-translational pathway. Insertion of PetD is dependent on the cpSRP54-cpFtsY-ALB3 protein complex. Possible participation of cpSRP43 proteins in this process cannot be excluded

Herein, we propose the cpSRP54-cpFtsY-ALB3-based transport mechanism (Fig. 8) that mediates the import of PetD protein into the thylakoid membranes. Furthermore, we show that the PetD integration into thylakoid membrane is post-translational and an SRP-dependent process that relies on cpSRP54-cpFtsY-ALB3-PetD complex formation. This machinery may require an atypical signal sequence, that we cannot identify using the current algorithms. Furthermore, we cannot exclude the possibility that cpSRP43 proteins do not “participate” in this process.

## Additional files

Additional file 1: Figure S1. The pT7CFE1-CHis which is optimized to use with the 1-Step Human In Vitro Protein Expression System. (PDF 165 kb)

Additional file 2: Figure S2. Western blot testing for cross-reaction of the anti-PetB, anti-cpSrp54 or ALB3 antibodies with proteins extracted from chloroplast. (PDF 195 kb)

Additional file 3: Figure S4. The Kyte–Doolittle hydropathy profile of the first 75 amino acids of pea PetD and ceQORH (Chloroplast Envelope Quinone Oxido-Reductase Homologue). (PDF 146 kb)

Additional file 4: Figure S5. MALDI-TOF mass spectra. (PDF 112 kb)

Additional file 5: Figure S6. Analyses of thylakoid membrane fractions and stroma after insertion of PetB or PsbW protein by spontaneous pathway. (PDF 121 kb)

Additional file 6: Table S1. Analysis of proteins co-immunoprecipitated with PetD after in vitro translation followed by posttranslational insertion into thylakoid membrane. (PDF 119 kb)

Additional file 7: Figure S7. Autoradiograph of isolated free and membrane bound ribosomes isolated during cell-free expression of PetD. (PDF 235 kb)

Additional file 8: Table S2. Analysis of proteins co-immunoprecipitated with PetD-cytochrome *b*_*6*_ complexes after in vitro translation followed by insertion into thylakoid membrane. (PDF 140 kb)

Additional file 9: Figures S8 and S9. Sequence alignment of CCB1 and CCB3 proteins. (PDF 122 kb)

Additional file 10: Figure S3. Sequence alignment of PetD protein. (PDF 351 kb)

## Abbreviations

Ab: antibody
BAEE: Na-benzoyl-arginine ethyl ester
BSA: bovine serum albumin
BSOCOES: *N*-hydroxysuccinade ester bis[2-(succinimidyloxycarbonyloxy) ethyl] sulfone
CHL: chlorophyll
cTP: chloroplast transit peptides
DDM: n-Dodecyl-β-D-maltoside
DTT: dithiothreitol
EGTA: aminopolycarboxylic acid
LHCB1: subtype light-harvesting chlorophyll a/b binding proteins
LHCPs: light-harvesting chlorophyll a/b-binding proteins
lTP: lumenal transfer peptide
MS: Q-TOF mass spectroscopy
mTP: mitochondrial targeting peptide
MW: molecular weight
PetA: cytochrome *f*
PetB: cytochrome *b*_*6*_
PetD: subunit IV
PMF: peptide mass fingerprints
PsbW: subunit W of PS II
PSI: Photosystem I
PSII: Photosystem II
RNCs: ribosome nascent chain complexes
SDS: sodium dodecyl sulphate
SDS-PAGE: sodium dodecyl sulphate polyacrylamide gel electrophoresis
Sec: secretory pathway
SP: secretory proteins
SPDP: N-succinimidyl-3-[2-pyridyldithio]propionate
SRP: signal recognition particle
TM: transmembrane
TMH: transmembrane helices
