# Supplementary material for: Chloroplast PetD protein: evidence for SRP/Alb3-dependent insertion into the thylakoid membrane
Source: BMC Plant Biol. 2017 Nov 21;17:213. doi: 10.1186/s12870-017-1176-2 (PMC5697057; doi:10.1186/s12870-017-1176-2)
Supplement: Supplementary file 1 — The pT7CFE1-CHis which is optimized to use with the 1-Step Human In Vitro Protein Expression System. (PDF 165 kb) [file 12870_2017_1176_MOESM1_ESM.pdf]

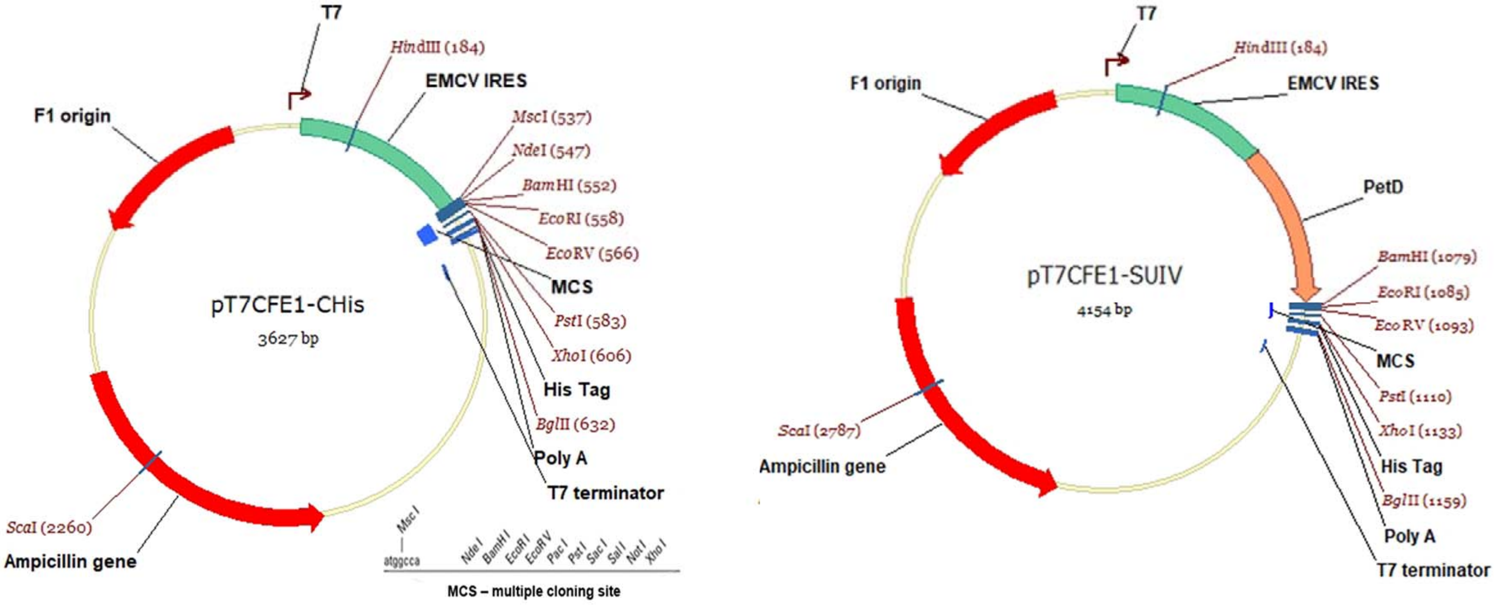

Figure S1. The pT7CFE1-CHis which is optimized to use with the 1-Step Human In Vitro Protein Expression System. *PetD* gene was cloned between Msc I and BamH I restriction sites using standard molecular technics. Resulting plasmid was named pT7CFE1-SUIV.
