# Supplementary material for: Chloroplast PetD protein: evidence for SRP/Alb3-dependent insertion into the thylakoid membrane
Source: BMC Plant Biol. 2017 Nov 21;17:213. doi: 10.1186/s12870-017-1176-2 (PMC5697057; doi:10.1186/s12870-017-1176-2)
Supplement: Supplementary file 2 — Western blot testing for cross-reaction of the anti-PetB, anti-cpSrp54 or ALB3 antibodies with proteins extracted from chloroplast. (PDF 195 kb) [file 12870_2017_1176_MOESM2_ESM.pdf]

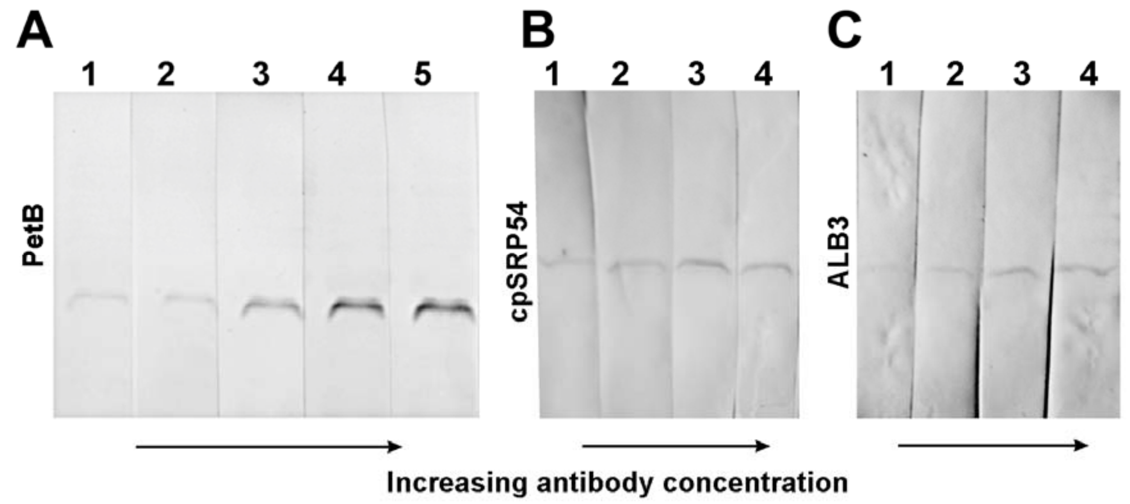

Figure S2. Western blot testing for cross-reaction of the anti-PetB, anti-cpSrp54 or ALB3 antibodies with proteins extracted from chloroplast. Trials were performed with various dilutions of purified antibody. To assess the specificity and cross-reactivity of these antibodies, on each path, an equal amount of protein preparation (20 μg) was applied. Antibodies initial concentrations were: 0.380 mg/ml (anti-PetB), 0.480 mg/ml (anti-cpSRP54), 0.313 mg/ml (anti-ALB3). Following dilutions were used for immunodetection: Lane 1. 64,000; lane 2, 32, 000; lane 3, 16,000; lane 8,000; lane 5, 4,000.
