# Supplementary material for: Chloroplast PetD protein: evidence for SRP/Alb3-dependent insertion into the thylakoid membrane
Source: BMC Plant Biol. 2017 Nov 21;17:213. doi: 10.1186/s12870-017-1176-2 (PMC5697057; doi:10.1186/s12870-017-1176-2)
Supplement: Supplementary file 3 — The Kyte–Doolittle hydropathy profile of the first 75 amino acids of pea PetD and ceQORH (Chloroplast Envelope Quinone Oxido-Reductase Homologue). (PDF 146 kb) [file 12870_2017_1176_MOESM3_ESM.pdf]

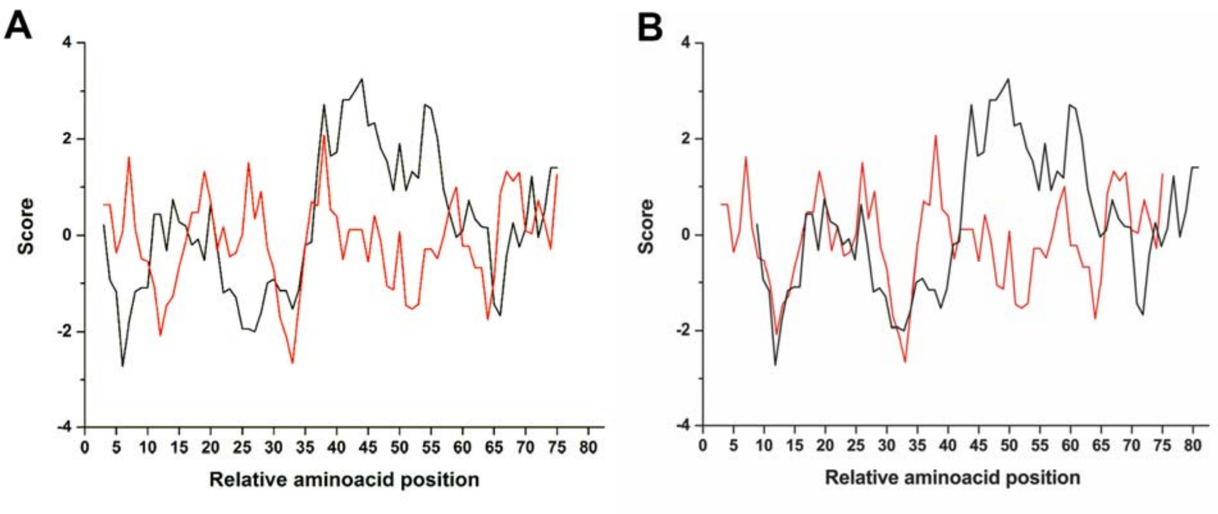

Figure S4. The Kyte–Doolittle hydropathy profile of the first 75 amino acids of pea PetD (black line) and ceQORH (Chloroplast Envelope Quinone Oxido-Reductase Homologue, (red line). **A:** A relative sequence numbering is given, with position 0 representing the first residue of the PetD, and the window size was set to 5 residues for sequences comparison. **B:** A relative sequence numbering is given, with position 0 representing the first residue of the ceQORH protein. PedD sequence is shifted by 8 residues towards C-terminus to show the likely similarity. Kyte–Doolittle hydropathy plots [1] were generated using an online tool from the ExPASy molecular biology server (http://www.expasy.org/tools/protscale.html).

1. Kyte J, Doolittle RF: A simple method for displaying the hydropathic character of a protein. *J Mol Biol* 1982, 157(1):105-132.
