# Supplementary material for: Chloroplast PetD protein: evidence for SRP/Alb3-dependent insertion into the thylakoid membrane
Source: BMC Plant Biol. 2017 Nov 21;17:213. doi: 10.1186/s12870-017-1176-2 (PMC5697057; doi:10.1186/s12870-017-1176-2)
Supplement: Supplementary file 4 — MALDI-TOF mass spectra. (PDF 112 kb) [file 12870_2017_1176_MOESM4_ESM.pdf]

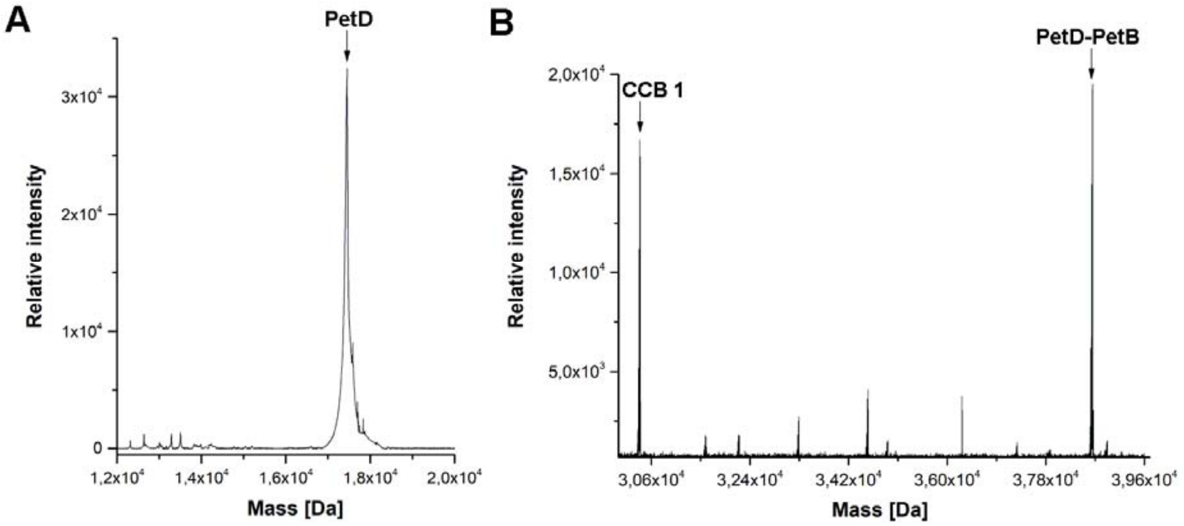


**Figure S5. MALDI-TOF mass spectra. A**: Mass spectrum of radiolabeled PetD isolated from thylakoid membrane followed by SDS-PAGE and MALDI-TOF. **B:** Mass spectrum of isolated from thylakoid membrane PetD and cytochrome $b_6$ crosslinked (SPDP) complex followed by immunoprecipitation with anibody against cytochrome $b_6$. The intensity (peak height on the y-axis) of the resulting MALDI spectrum is not quantitative, only the mass is obtained.
