# Supplementary material for: Chloroplast PetD protein: evidence for SRP/Alb3-dependent insertion into the thylakoid membrane
Source: BMC Plant Biol. 2017 Nov 21;17:213. doi: 10.1186/s12870-017-1176-2 (PMC5697057; doi:10.1186/s12870-017-1176-2)
Supplement: Supplementary file 5 — Analyses of thylakoid membrane fractions and stroma after insertion of PetB or PsbW protein by spontaneous pathway. (PDF 121 kb) [file 12870_2017_1176_MOESM5_ESM.pdf]

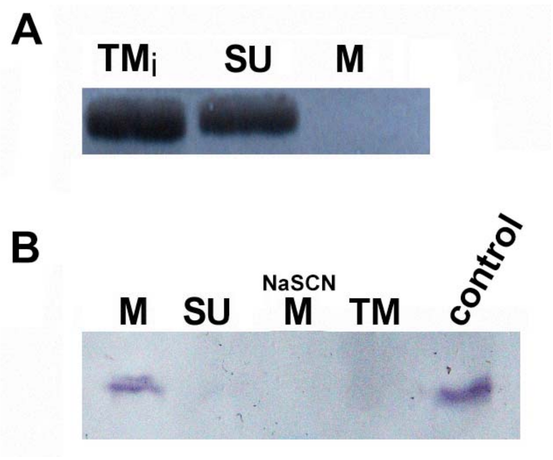

Figure S6. Analyses of thylakoid membrane fractions and stroma after insertion of PetB or PsbW protein by spontaneous pathway. A: Autoradiograph of thylakoid membrane fractions after integration of radiolabeled PetB in the presence of thylakoid membrane, supernatant (SU) and membrane pellet (M) after fractionation respectively. Total translation mixture was used as a control (TM_i); **B:** Western blot analysis of the stroma and membrane fraction after PsbW protein insertion [1]. Antiserum directed against the biotin was used. Free biotin from membrane extract was removed by dialysis. Covalently bound biotin by membrane protein was blocked by specific biotin blocking step before incubation with the primary antibody (Endogenous Biotin-Blocking Kit, Thermo Scientific). supernatant (SU) and membrane pellet (M) after fractionation were analysed before and after washing with chaotropic agent (NaSCN). The reaction assay after PsbW insertion was used as a control.

1. Kroliczewski J, Piskozub M, Bartoszewski R, Kroliczewska B: ALB3 Insertase Mediates Cytochrome b_6 Co-translational Import into the Thylakoid Membrane. *Sci Rep* 2016, 6:34557.
