# Supplementary material for: Chloroplast PetD protein: evidence for SRP/Alb3-dependent insertion into the thylakoid membrane
Source: BMC Plant Biol. 2017 Nov 21;17:213. doi: 10.1186/s12870-017-1176-2 (PMC5697057; doi:10.1186/s12870-017-1176-2)
Supplement: Supplementary file 6 — Analysis of proteins co-immunoprecipitated with PetD after in vitro translation followed by posttranslational insertion into thylakoid membrane. (PDF 119 kb) [file 12870_2017_1176_MOESM6_ESM.pdf]

Table S1. Analysis of proteins co-immunoprecipitated with PetD after *in vitro* translation followed by posttranslational insertion into thylakoid membrane. Immunoprecipitation of PetD complexes using an antibody against ALB3. Bound proteins were directly analysed by ESI-MS/MS with fingerprints analysis.

Annotation in database	[d]Protein	[b]Peptides	[d]Score	Expect value	[a]Total Score	[c]Queries matched
AAD41889	PetD	MGVTKKPDLTDPVLR<br>LLGVLLMVSVPAGLLTVPFLENVNK<br>SLTLGLF	284<br>319<br>147	6.8e-08<br>2.9e-08<br>3.9e-05	750	12
NP_001189626	ALBINO3 (ALB3)	ALQQRYAGNQER<br>YAGNQER	269<br>204	5.9e-07<br>1.7e-05	473	8
AAC64109	cpSRP54	LDGDSRGGAALSVK<br>EVSGKPIKLVGR<br>TEQQVSQLVAQLFQMR	205<br>134<br>178	2.3e-07<br>1.3e-05<br>0.0009	517	9
NP_566056	cpFtsY	DALKESVLEMLAK<br>KPAVIMIVGVNGGGK<br>TGCEIVVAEGDK<br>LHTNYSLMEELIACK	129<br>88<br>94<br>87	1.2e-05<br>5.3e-06<br>0.00023<br>8.2e-06	398	14
ACJ85404	unknow	EGVEVTQIVR<br>EGQPLGIAVTLR	72<br>45	9.3e-05<br>0.016	117	3
CAA33264	GAPDH	TFAEEVNEAFR<br>VPTPNVSVVDLVVQVSK<br>ELGIDLVIEGTGVFVDR	83<br>110<br>73	3.4e-09<br>5.1e-07<br>7e-06	266	10
AAM98365	Rubisco, large subunit	ELGVPIVMHDYLTGGFTAN	59	0.0018	59	2
O22229	NTR3	EGTSNTSVEGVFAAGDVQDHEWR	48	0.0083	48	2
1EKJ_A	Beta Carbonic Anhydrase	YAGTGAAIEYAVLHLK<br>EAVNASLGNLLTYPFVR	41<br>72	6.4e-06<br>0.0027	113	3
P27520	LHCB	ELEVIHSR<br>SAPESIWYGPDRPK	59<br>58	0.0011<br>0.0015	117	4
BAC10691	sAPX	IPMKYGR<br>SGWGKPETK	21<br>36	4.3e-05<br>0.0062	57	3
AED90364	FER1	FMEYQNQR	36	0.0002	46	2

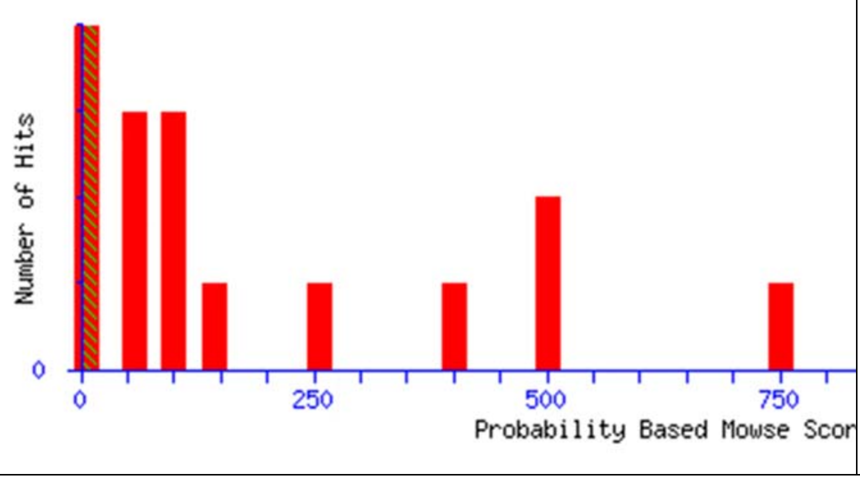

Probability Based Mowse Score

Ions score is -10*Log(P), where P is the probability that the observed match is a random event. Individual ions scores > 41 indicate identity or extensive homology (p<0.05).

Protein scores are derived from ions scores as a non-probabilistic basis for ranking protein hits.
