# Supplementary material for: Chloroplast PetD protein: evidence for SRP/Alb3-dependent insertion into the thylakoid membrane
Source: BMC Plant Biol. 2017 Nov 21;17:213. doi: 10.1186/s12870-017-1176-2 (PMC5697057; doi:10.1186/s12870-017-1176-2)
Supplement: Supplementary file 7 — Autoradiograph of isolated free and membrane bound ribosomes isolated during cell-free expression of PetD. (PDF 235 kb) [file 12870_2017_1176_MOESM7_ESM.pdf]

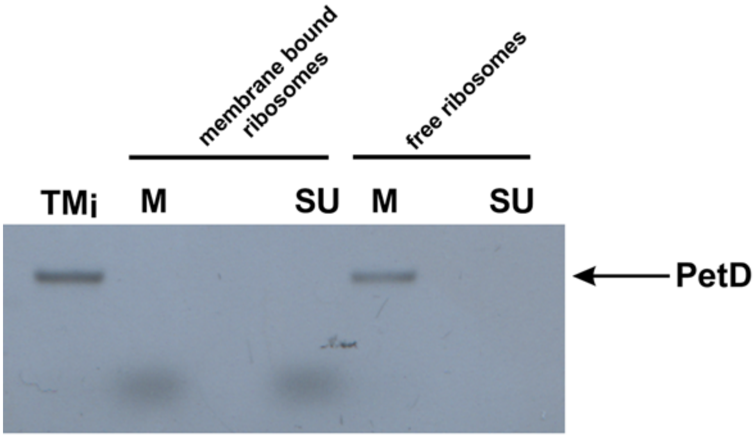

Figure S7. Autoradiograph of isolated free and membrane bound ribosomes isolated during cell-free expression of PetD. Free polysomes were prepared by the method of Friemann and Hachtel [1] but thylakoid-bound ribosomes were detached according to Margulies, et al. [2]. Total translation mixture (TM_i) was used as a control. M -membrane pellet; SU- supernatant (SU).

1. Friemann A, Hachtel W: Chloroplast messenger RNAs of free and thylakoid-bound polysomes from Vicia faba L. *Planta* 1988, 175(1):50-59.
2. Margulies MM, Tiffany HL, Hattori T: Photosystem I reaction center polypeptides of spinach are synthesized on thylakoid-bound ribosomes. *Arch Biochem Biophys* 1987, 254(2):454-461.
