# Supplementary material for: Chloroplast PetD protein: evidence for SRP/Alb3-dependent insertion into the thylakoid membrane
Source: BMC Plant Biol. 2017 Nov 21;17:213. doi: 10.1186/s12870-017-1176-2 (PMC5697057; doi:10.1186/s12870-017-1176-2)
Supplement: Supplementary file 8 — Analysis of proteins co-immunoprecipitated with PetD-cytochrome b 6 complexes after in vitro translation followed by insertion into thylakoid membrane. (PDF 140 kb) [file 12870_2017_1176_MOESM8_ESM.pdf]

Table S2. Analysis of proteins co-immunoprecipitated with PetD-cytochrome b_6 complexes after *in vitro* translation followed by insertion into thylakoid membrane. Immunoprecipitation of complexes using an antibody against cytochrome b_6. Bound proteins were directly analysed by ESI-MS/MS with fingerprints analysis.

[e]PetD/PetB complex						
Annotation in database	[d]Protein	[b]Peptides	[d]Score	Expect value	[a]Total Score	[e]Queries matched
AIK21467	PetB	LEIQAIADDITSK<br>VYLTGGFK<br>IVTGVPDAIPVIGSSVVELLR	182<br>130<br>158	3.7e-06<br>2.1e-07<br>0.00011	480	9
AAD41889	PetD	KPDLTDPVLRAK<br>LLGVLLMVSVPAGLLTVPFLENVNK<br>FQNPFR<br>RPVATTVFLIGTVVALWLGIGATLPIEK	118<br>163<br>77<br>270	2.7e-07<br>6.9e-06<br>1.5e-05<br>1.2e-08	628	14
Pisum_sativum_v1_Contig2650 [6]	CCB1	IVNKTFVK<br>LLDEVGNKAPNQVAGEVLSFFTR<br>EDGTLSEIVVQGDDQQVEQMRK	48<br>74<br>103	0.00024<br>2.3e-05<br>1.7e-09	225	11
Pisum sativum_csfl_reftrans V1_0082495 [6]	CCB3	LMILADLDPATAK<br>FPYVIAYAPTEPLLVPTRK	47<br>52	3.8e-07<br>0.00033	99	5
Q9FNM5	GUFP	VIASEALSAIR	49	0.0079	43	3
CAA33264	GAPDH	TFAEEVNEAFR<br>ELGIDLVIEGTGVFVDR	68<br>49	2.7e-06<br>0.00071	117	4

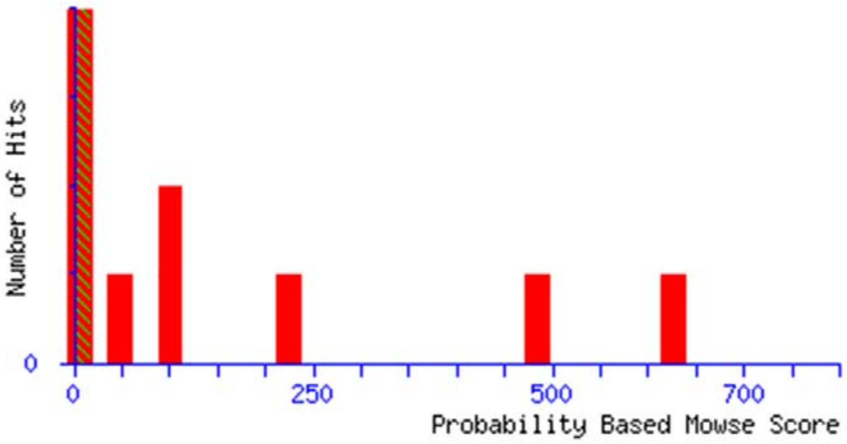

Probability Based Mowse Score

Ions score is -10*Log(P), where P is the probability that the observed match is a random event. Individual ions scores > 41 indicate identity or extensive homology (p<0.05). Protein scores are derived from ions scores as a non-probabilistic basis for ranking protein hits.

Legend for both tables:

[a]Others proteins with higher scores than 40 were not observed after cross-linking.

[b]Peptide identifications were accepted if they could be established at greater than 80.0% probability. To calculate total score, only the individual ion scores with an expected value less than 0.05 were chosen for the identified protein. We chose only proteins with unique queries. The number of matches of MS/MS spectra that uniquely match the accession and were not shared with other accessions were identified. These matched spectra pass the minimal criteria for ion score and have a false positive rate of less 1%. Furthermore, the analysis resulted in the identification of several hundred of peptides that were impossible to assign to a specific protein. Therefore, the final analysis score cut off was set at 20 to eliminate low-score peptides, and 40 to eliminate low-score proteins. Individual ions score > 41 indicate identity or extensive homology (p < 0.05). To calculate total score, the individual ion scores with the expected value

lower than 0.05 were chosen for the identified peptide (http://www.matrixscience.com/help/scoring_help.html) [1].
[c]Total number of queries: 1124
[d]Proteins with total score less than 40 and peptides score less than 20:129
[e]Total number of queries: 243

Search Parameters

Type of search: MS/MS Ion Search
Enzyme: Trypsin
Fixed modifications: Carbamidomethyl (C)
Variable modifications: Oxidation (M), Carbamidomethyl (K)
Mass values: Monoisotopic
Protein Mass: Unrestricted
Peptide Mass Tolerance: ± 40 ppm
Fragment Mass Tolerance: ± 0.8 Da
Max Missed Cleavages: 1
Instrument type: ESI-TRAP
Database: NCBInr
Taxonomy: Viridiplantae (Green Plants) (730741 sequences)

1. Perkins DN, Pappin DJ, Creasy DM, Cottrell JS: Probability-based protein identification by searching sequence databases using mass spectrometry data. Electrophoresis 1999, 20(18):3551-3567.
