# Supplementary material for: Chloroplast PetD protein: evidence for SRP/Alb3-dependent insertion into the thylakoid membrane
Source: BMC Plant Biol. 2017 Nov 21;17:213. doi: 10.1186/s12870-017-1176-2 (PMC5697057; doi:10.1186/s12870-017-1176-2)
Supplement: Supplementary file 9 — Sequence alignment of CCB1 and CCB3 proteins. (PDF 122 kb) [file 12870_2017_1176_MOESM9_ESM.pdf]

```
                                        1                                      40
Arabidopsis thaliana  CCB1    (1) ---MATKLISPPLSCPWVTSREVIIKGLPRRRREWMVTKR
     Spinacia oleracea CCB1    (1) MAANLFSPLSCYCSISNYKTRENYLSNQNHNQHHPFAANL
     Nicotiana tabacum CCB1    (1) -----MAAKLLLSPLSLPSSFLSTREFQHHHRHLCLRTKP
              Zea mays CCB1    (1) MDATTAVAARRLLLLPLRAQPPAPRGAAPATANPWCRSVP
        Pisum sativum CCB1    (1) ------------------------KLHPWNHQHHLPTPKP
                                   41                                      80
Arabidopsis thaliana  CCB1   (38) NR-VSAVTAMIVEPLSVVSSSAIQIHQWWEQNPNSLLLMT
     Spinacia oleracea CCB1   (41) KAKRVVKMGASVEEMSISSELATDLANHLHENP-SSVILM
     Nicotiana tabacum CCB1   (36) RNRFTVHAFTDVLFTAATTSAPLLVLDQFQENPSSLFSIA
              Zea mays CCB1   (41) RR------------RRVRAAPARASLDRAAVLLDTAAAVA
        Pisum sativum CCB1   (38) EK----------LRLSLHESAEFITQQRTTTKPQFSFLAY
                                   81                                     120
Arabidopsis thaliana  CCB1   (77) EATGGYSLASYYTSLGLFVISVPGLWSLIKRSVKSKIVRK
     Spinacia oleracea CCB1   (80) AESVGYSVASYYTSLGLFVISVPGLWSLIKRSVKSKIVKK
     Nicotiana tabacum CCB1   (76) AADSGYSVASYYTSLGLFVISVPGLWSLIKRSVKSKIVQK
              Zea mays CCB1   (69) NGGTGYSQASYYTSLGLFVLSVPGLWSLIKRSVKSKIVQK
        Pisum sativum CCB1   (68) RDCWGYSRASYYTSLGLFVISVPGLWSLVKRSVKSKIVNK
                                   121                                    160
Arabidopsis thaliana  CCB1  (117) TFVVNDVKK---EPKQVAGEILSFFTRKNFNITDRGETIT
     Spinacia oleracea CCB1  (120) TYVQGDEIK--KAPNQVAGEVLSYFTRNNFTVTDRGETIT
     Nicotiana tabacum CCB1  (116) TFVKEGIEEGKKAANQVAGEILSFFTRNNFTVLDRGETIT
              Zea mays CCB1  (109) TFVKDEGQS--MAPNQVAGEILSFFTRNNFTVSDRGEVIT
        Pisum sativum CCB1  (105) TFVKLLDEVGNKAPNQVAGEVLSFFTRNNFSVVDRGETIT
                                   161                                    200
Arabidopsis thaliana  CCB1  (154) FEGKMVPSRGQAALLTFCTCISLASVGLVLTITVPDFGNN
     Spinacia oleracea CCB1  (158) FEGTMLPSRGQAALLTFCTCISLASVALVLTITFPDVGNN
     Nicotiana tabacum CCB1  (156) FEGMMVPSRGQAALLTFCTCISLGSVALVLTITVPDVGNN
              Zea mays CCB1  (147) FEGTMIPNRGQAALLTFCTCISLGSVGLVLSIAVPEGGNN
        Pisum sativum CCB1  (145) FEGVMVPSRGQAALLTFCTCISLASVSLVLTITVPDVGNN
                                   201                                    240
Arabidopsis thaliana  CCB1  (194) WFFIILLSPLAGVYYWKKASRKEEIKVKMMVGSKGRLDEI
     Spinacia oleracea CCB1  (198) WFYIIALSPLAGAYYWKRATRKEQIKIKMVVRENGTLSEI
     Nicotiana tabacum CCB1  (196) WFWIIALSPLAGVYYWTRASRKEQIKVKMTVGDDGSLSEI
              Zea mays CCB1  (187) WFWLMTLSPLAGAYYWTKASRKEEIKVKMILSDDGNVSEI
        Pisum sativum CCB1  (185) WFGLTILSPLAGAYYWTRASRKEQIKVKMTVKEDGTLSEI
                                   241                                    280
Arabidopsis thaliana  CCB1  (234) VVQGDDVQVEEMRKELQLNEKGMVYVKGLFERSS------
     Spinacia oleracea CCB1  (238) VVQGDDQQVEQMRKELQLNEKGMVYVKGILER--------
     Nicotiana tabacum CCB1  (236) VVQGDDQEVEKMRKELQLSEKGMVYVKGLLER--------
              Zea mays CCB1  (227) LVQGDDVQVEQMRKDLKFSEKGMVYVKGIFET--------
        Pisum sativum CCB1  (225) VVQGDDQQVEQMRKELKFSEKGMVYVKGLFET-LVFIIWL
                                   281
```

Figure S8. Sequence alignment of CCB1 protein. Fully conserved residues are shaded in yellow, similar residues are shaded in green. The sequences from pea were aligned using MAFFT version 7 [1]. NCBI database: Arabidopsis thaliana (NP_566797), Spinacia oleracea (KNA04171), Nicotiana tabacum (XP_016507972.1), Zea mays (AQK57718). Cool Season Food Legume Genome Database: Pisum_sativum_v1_Contig2650 [2].

```
                                   1                                      40
Arabidopsis thaliana CCB3    (1) ---------------------------------MTTVTTS
   Nicotiana tabacum CCB3    (1) ---------MAISSLIPSPPQSIGYPAALLHPHTNGTPCD
   Spinacia oleracea CBB3    (1) ----------------------------------------
            Zea mays CCB3    (1) --MEASLLVAPKPSMPRTIFSARRAGRSWRCLVVAATIKQ
       Pisum sativum CCB3    (1) LHVREGSLLI-LLSC-PLTMATQSYLVNLNSFHTGITAPN
                                 41                                     80
Arabidopsis thaliana CCB3    (8) FVSFSPALMIFQKKSRRSSPNFRNRSTSLPIVSATLSHIE
   Nicotiana tabacum CCB3   (32) YGNFLIPMRRKN-IQSCRDTKFRACSYLDGSVSATIELVQ
   Spinacia oleracea CBB3    (1) ----------------------------------------
            Zea mays CCB3   (39) QLGTRSESVDVDAPVEHLAPPSKATTGARGGLLAAVATTT
       Pisum sativum CCB3   (39) PSIFKPPNFGFGIWKSLKHPTRRNADGSSRIMQCCSCYTE
                                 81                                    120
Arabidopsis thaliana CCB3   (48) E-AATTTN---------------LIRQTNSISESLRNISL
   Nicotiana tabacum CCB3   (71) SNGNVPISPLNSE-EVVKVLASSAIETSDASLPLMQRLVL
   Spinacia oleracea CBB3    (1) -----------------------MLETSHIISELKQNLLI
            Zea mays CCB3   (79) SSCTAVLPCHALSGAGAVES-GPWWWTSPLDTLYRAAAVL
       Pisum sativum CCB3   (79) VGAASALKCLDLDTNLSSLNLNIPETLHASTDFMTRLMIL
                                 121                                   160
Arabidopsis thaliana CCB3   (72) ADLDPGTAKLAIGILGPALSAFGFLFILRIVMSWYPKLPV
   Nicotiana tabacum CCB3  (110) LDLDPATAKLAISFLGPFLSAFSFLFILRIVMSWYPKLPV
   Spinacia oleracea CBB3   (18) LDLDPATAKVAIPFIGPFLSAFSFLFIARIVMSWYPKLPV
            Zea mays CCB3  (118) GDLDPATARAVAGVAGPVLSAFGFLFILRIVMSWYPRLPV
       Pisum sativum CCB3  (119) ADLDPATAKFAIGFLGPFLSAFGFLFIARIVMSWYPKLPV
                                 161                                   200
Arabidopsis thaliana CCB3  (112) DKFPYVLAYAPTEPILVQTRKVIPPLAGVDVTPVVWFGLV
   Nicotiana tabacum CCB3  (150) GEFPYVIAYAPTEPILGATRKLIPPLAGVDVTPVVWFGLV
   Spinacia oleracea CBB3   (58) GKFPYVLAYAPTEPILAPTRKVIPPLGGVDVTPVVWFGLL
            Zea mays CCB3  (158) TEFPYVVAYAPTEPFLAVTRRVIPPLGGVDVTPVVWFGLV
       Pisum sativum CCB3  (159) GKFPYVIAYAPTEPLLVPTRKVIPPLAGVDVTPVVWFGLL
                                 201                                   240
Arabidopsis thaliana CCB3  (152) SFLSEILVGPQGLLVLVSQQQVN-----------------
   Nicotiana tabacum CCB3  (190) SFINEILLGPQGLLVLLSQKQV------------------
   Spinacia oleracea CBB3   (98) SFLNEILVGPQGLLVLLSQQMSS-----------------
            Zea mays CCB3  (198) SFASEILVGPQGLLVLLSQQR-------------------
       Pisum sativum CCB3  (199) SFLNEILVGPQGLLVLLSQQVN-FAHLMCLMTLINLHIWC
                                 241                                   280
```

Figure S9. Sequence alignment of CCB3 protein. Fully conserved residues are shaded in yellow, similar residues are shaded in green. The sequences from pea were aligned using MAFFT version 7 [1]. NCBI database: Arabidopsis thaliana (NP_198461), Spinacia oleracea (KNA17454), Nicotiana tabacum (XP_016463713), Zea mays (AQK98046). Cool Season Food Legume Genome Database: Pisum sativum_csfl_reftransV1_0082495 [2].

1. Katoh K, Standley DM: MAFFT Multiple Sequence Alignment Software Version 7: Improvements in Performance and Usability. *Mol Biol Evol* 2013, 30(4):772-780.
2. Humann J, Jung S, Zheng P, Cheng C-H, Lee T, Frank M, McGaughey D, Scott K, Yu J, Ficklin S *et al*: Cool Season Food Legume Genome Database: An up-to-date resource enabling genetics, genomics and breeding research in pea, lentil, faba bean and chickpea. In: *Plant & Animal Genome Conference XXIV: 2016; San Diego, CA, USA.*; 2016.
