# Supplementary material for: Chloroplast PetD protein: evidence for SRP/Alb3-dependent insertion into the thylakoid membrane
Source: BMC Plant Biol. 2017 Nov 21;17:213. doi: 10.1186/s12870-017-1176-2 (PMC5697057; doi:10.1186/s12870-017-1176-2)
Supplement: Supplementary file 10 — Sequence alignment of PetD protein. (PDF 351 kb) [file 12870_2017_1176_MOESM10_ESM.pdf]

```
 Synechococcus sp. RCC307            (1)  MHILKEPDLNDPKLRAKLAKGMGHNYYGEPAWPNDLLYIFP
 Synechococcus sp. WH 7803           (1)  -------------MRAKLAKGMGHNYYGEPAWPNDLLYIFP
 Synechococcus sp. WH 8102           (1)  MHILKKPDLSDPKMRAKLAKGMGHNYYGEPAWPNDLLYIFP
 Synechococcus sp. CC9311            (1)  MHILKKPDLTDPKLRAKLAKGMGHNYYGEPAWPNDLLYIFP
 Synechococcus sp. CC9605            (1)  MHILKKPDLSDPKMRAKLAKGMGHNYYGEPAWPNDLLYIFP
 Synechococcus sp. CC9902            (1)  MHILKKPDLSDPKMRAKLAKGMGHNYYGEPAWPNDLLYIFP
 Synechococcus elongatus PCC 6301    (1)  MSILKKPDLSDPILRQKLAKGMGHNYYGEPAWPNDLLYMFP
 Synechococcus elongatus PCC 7942    (1)  MSILKKPDLSDPILRQKLAKGMGHNYYGEPAWPNDLLYMFP
 Synechocystis sp. PCC 6803          (1)  MSIIKKPDLSDPDLRAKLAKGMGHNYYGEPAWPNDILYMFP
 Chlamydomonas reinhardtii           (1)  MSVTKKPDLSDPVLKAKLAKGMGHNTYGEPAWPNDLLYMFP
 Porphyra purpurea                   (1)  MSILKKPDLTDPKLRAKLAKGMGHNYYGEPAWPNDLLYVFP
 Marchantia polymorpha               (1)  MGVTKKPDLSDPILRAKLAKGMGHNYYGEPAWPNDLLYIFP
 Pinus koraiensis                    (1)  MGVTKKPDLNDPVLRAKLAKGMGHNYYGEPAWPNDLSYIFP
 Oenothera elata subsp. Hookeri      (1)  MGVTKKPDLNDPVLRAKLAKGMGHNYYGEPAWPNDLLYIFP
 Oryza sativa                        (1)  MGVTKKPDLNDPVLRAKLAKGMGHNYYGEPAWPNDLLYIFP
 Arabidopsis thaliana                (1)  MGVTKKPDLNDPVLRAKLAKGMGHNYYGEPAWPNDLLYIFP
 Spinacia oleracea                   (1)  MGVTKKPDLNDPVLRAKLAKGMGHNYYGEPAWPNDLLYIFP
 Lactuca sativa                      (1)  MPITKKPDLNDPVLRAKLAKGMGHNYYGEPAWPNDLLYIFP
 Panax ginseng                       (1)  MGVTKKPDLNDPVLRAKLAKGMGHNYYGEPAWPNDLLYIFP
 Populus trichocarpa                 (1)  MGVTKKPDLNDPVLRAKLAKGMGHNYYGEPAWPNDLLYIFP
 Zea mays                            (1)  MGVTKKPDLNDPVLRAKLAKGMGHNYYGEPAWPNDLLYIFP
 Nicotiana tabacum                   (1)  MGVTKKPDLNDPVLRAKLAKGMGHNYYGEPAWPNDLLYIFP
 Consensus                           (1)  M VTKKPDLNDPVLRAKLAKGMGHNYYGEPAWPNDLLYIFP

                                     (42) 42      50        60        70         82
 Synechococcus sp. RCC307            (42) VVILGTIACVVALAVLDPAMLADKADPFATPLEILPEWYLY
 Synechococcus sp. WH 7803           (29) VVILGTIACVVGLAVLDPAMLGDKADPFATPLEILPEWYLY
 Synechococcus sp. WH 8102           (42) VVILGTIACIVGLAVLDPAMLADKADPFATPLEILPEWYLY
 Synechococcus sp. CC9311            (42) VVILGTIACIVGLSVLDPAMLGDKADPFATPLEILPEWYLY
 Synechococcus sp. CC9605            (42) VVILGTIACVVGLAVLDPAMLADKADPFATPLEILPEWYLY
 Synechococcus sp. CC9902            (42) VVILGTIACVVGLAVLDPAMLADKADPFATPLEILPEWYLY
 Synechococcus elongatus PCC 6301    (42) VVILGTIACLTGLAVLDPALVGEPADPFATPLEILPEWYLY
 Synechococcus elongatus PCC 7942    (42) VVILGTIACLTGLAVLDPALVGEPADPFATPLEILPEWYLY
 Synechocystis sp. PCC 6803          (42) ICILGALGLIAGLAILDPAMIGEPADPFATPLEILPEWYLY
 Chlamydomonas reinhardtii           (42) VVILGTFACVIGLSVLDPAAMGEPANPFATPLEILPEWYFY
 Porphyra purpurea                   (42) VVILGTIACSIGLAILEPSSLGEKSNPFATPLEILPEWYFF
 Marchantia polymorpha               (42) VVILGTIACTVGLAVLEPSMIGEPANPFATPLEILPEWYFF
 Pinus koraiensis                    (42) VVILGTIACTIGLAVLEPSMIGEPANPFATPLEILPEWYLF
 Oenothera elata subsp. Hookeri      (42) VVILGTIACNVGLAILEPSMLGEPADPFATPLEILPEWYFF
 Oryza sativa                        (42) VVILGTIACNVGLAVLEPSMIGEPADPFATPLEILPEWYFF
 Arabidopsis thaliana                (42) VVILGTIACNVGLAVLEPSMIGEPADPFATPLEILPEWYFF
 Spinacia oleracea                   (42) VVILGTIACNVGLAVLEPSMIGEPADPFATPLEILPEWYFF
 Lactuca sativa                      (42) VVILGTIACNVGLAVLEPSMIGEPADPFATPLEILPEWYFF
 Panax ginseng                       (42) VVILGTIACNVGLAVLEPSMIGEPADPFATPLEILPEWYFF
 Populus trichocarpa                 (42) VVILGTIACNVGLAVLEPSMIGEPADPFATPLEILPEWYFF
 Zea mays                            (42) VVILGTIACNVGLAVLEPSMIGEPADPFATPLEILPEWYFF
 Nicotiana tabacum                   (42) VVILGTIACNVGLAVLEPSMIGEPADPFATPLEILPEWYFF
 Consensus                           (42) VVILGTIAC VGLAVLEPSMIGEPADPFATPLEILPEWYFF
```

```
                                        (83)  83     90        100       110         123
           Synechococcus sp. RCC307     (83)  PVFQILRVVP--------NKLLGIALQTMIPLGLMLVPFIE
           Synechococcus sp. WH 7803    (70)  PVFQILRVVP--------NKLLGIALQTLIPLGLMLVPFIE
           Synechococcus sp. WH 8102    (83)  PVFQILRVVP--------NKLLGIALQTLVPLGLMLVPFIE
           Synechococcus sp. CC9311     (83)  PVFQILRVVP--------NKLLGIALQTLVPLGLMLIPFIE
           Synechococcus sp. CC9605     (83)  PVFQILRVVP--------NKLLGIALQTLVPLGLMLVPFIE
           Synechococcus sp. CC9902     (83)  PVFQILRVVP--------NKLLGIALQTLVPLGLMLVPFIE
  Synechococcus elongatus PCC 6301      (83)  PVFQILRIVP--------NKLLGIVLQSMIPLGLIAIPFIE
  Synechococcus elongatus PCC 7942      (83)  PVFQILRIVP--------NKLLGIVLQSMIPLGLIAIPFIE
           Synechocystis sp. PCC 6803   (83)  PTFQILRILP--------NKLLGIAGMAAIPLGLMLVPFIE
           Chlamydomonas reinhardtii    (83)  PVFQILRVVP--------NKLLGVLLMAAVPAGLITVPFIE
                   Porphyra purpurea    (83)  PTFNLLRVIP--------NKLLGVLSMAAVPAGLLTVPFIE
               Marchantia polymorpha    (83)  PVFQILRTVP--------NKLLGVLLMAAVPAGLLTVPFLE
                   Pinus koraiensis     (83)  PVFQILRTVPNQILRTVPNKLLGVLLMASVPAGSLTVPFLE
     Oenothera elata subsp. Hookeri     (83)  PVFQILRTVP--------NKLLGVLLMVSVPSGLLTVPFLE
                        Oryza sativa    (83)  PVFQILRTVP--------NKLLGVLLMVSVPTGLLTVPFLE
                Arabidopsis thaliana    (83)  PVFQILRTVP--------NKLLGVLLMVSVPAGLLTVPFLE
                   Spinacia oleracea    (83)  PVFQILRTVP--------NKLLGVLLMASVPAGLLTVPFLE
                      Lactuca sativa    (83)  PVFQILRTVP--------NKLLGVLLMVSVPAGLLTVPFLE
                       Panax ginseng    (83)  PVFQILRTVP--------NKLLGVLLMVSVPAGLLTVPFLE
                 Populus trichocarpa    (83)  PVFQILRTVP--------NKLLGVLLMVSVPAGLLTVPFLE
                            Zea mays    (83)  PVFQILRTVP--------NKLLGVLLMVSVPTGLLTVPFLE
                   Nicotiana tabacum    (83)  PVFQILRTVP--------NKLLGVLLMVSVPAGLLTVPFLE
                           Consensus    (83)  PVFQILRTVP        NKLLGVLLM SVPAGLLTVPFIE

                                       (124)  124    130       140       150         164
           Synechococcus sp. RCC307    (116)  SFNKFQNPFRRPVAMAVFLFGTAFTIYLGIGAALPIDKSLT
           Synechococcus sp. WH 7803   (103)  SFNKFQNPFRRPVAMAVFLFGTVTTIYLGIGAALPIDKSLT
           Synechococcus sp. WH 8102   (116)  SFNKFQNPFRRPVAMTVFLFGTVTTIYLGIGAALPIDKSLT
           Synechococcus sp. CC9311    (116)  SFNKFQNPFRRPIAMAVFLFGTATTIYLGIGAAMPIDKSLT
           Synechococcus sp. CC9605    (116)  SFNKFQNPFRRPVAMTVFLTGTLVTIYLGIGAALPIDKSLT
           Synechococcus sp. CC9902    (116)  SFNKFQNPFRRPVAMTVFLFGTFTTIYLGIGAAMPIDKSLT
  Synechococcus elongatus PCC 6301     (116)  SVNKFQNPFRRPIATAVFLFGTVFTLYLGIGAALPIDKSLT
  Synechococcus elongatus PCC 7942     (116)  SVNKFQNPFRRPIATAVFLFGTVFTLYLGIGAALPIDKSLT
           Synechocystis sp. PCC 6803  (116)  SVNKFQNPFRRPIAMTVFLFGTAAALWLGAGATFPIDKSLT
           Chlamydomonas reinhardtii   (116)  SINKFQNPYRRPIATILFLLGTLVAVWLGIGSTFPIDISLT
                   Porphyra purpurea   (116)  NVNKFQNPFRRPIATTIFLISTVITIWLGIGATMPINNAIT
               Marchantia polymorpha   (116)  NVNKFQNPFRRPVATTVFLIGTVVALWLGIGAALPIDKSLT
                   Pinus koraiensis    (124)  NVNQFQNPFRRPVATTVSLIGTAVALWLGIGAALPIDESLT
     Oenothera elata subsp. Hookeri    (116)  NVNKFQNPFRRPVATTVFLIGTVVALWLGIGATLPIDKSLT
                        Oryza sativa   (116)  NVNKFQNPFRRPVATTVFLIGTAVALWLGIGATLPIEKSLT
                Arabidopsis thaliana   (116)  NVNKFQNPFRRPVATTVFLIGTAAALWLGIGATLPIDKSLT
                   Spinacia oleracea   (116)  NVNKFQNPFRRPVATTVFLVGTVVALWLGIGATLPIDKSLT
                      Lactuca sativa   (116)  NVNKFQNPFRRPVATTVFLIGTAVALWLGIGATLPIDKSLT
                       Panax ginseng   (116)  NVNKFQNPFRRPVATTVFLIGTAVALWLGIGATLPIDKSLT
                 Populus trichocarpa   (116)  NVNKFQNPFRRPVATTVFLIGTVVALWLGIGATLPIDKSLT
                            Zea mays   (116)  NVNKFQNPFRRPVATTVFLIGTAVALWLGIGATLPIDKSLT
                   Nicotiana tabacum   (116)  NVNKFQNPFRRPVATTVFLIGTAVALWLGIGATLPIDKSLT
                           Consensus   (124)  NVNKFQNPFRRPVATTVFLIGTVVALWLGIGATLPIDKSLT
```

	(165)	165 170 189
Synechococcus sp. RCC307	(157)	LGLF---------------------
Synechococcus sp. WH 7803	(144)	LGLF---------------------
Synechococcus sp. WH 8102	(157)	LGLF---------------------
Synechococcus sp. CC9311	(157)	LGLF---------------------
Synechococcus sp. CC9605	(157)	LGLF---------------------
Synechococcus sp. CC9902	(157)	LGLF---------------------
Synechococcus elongatus PCC 6301	(157)	LGLF---------------------
Synechococcus elongatus PCC 7942	(157)	LGLF---------------------
Synechocystis sp. PCC 6803	(157)	LGLF---------------------
Chlamydomonas reinhardtii	(157)	LGLF---------------------
Porphyra purpurea	(157)	LGLF---------------------
Marchantia polymorpha	(157)	LGLF---------------------
Pinus koraiensis	(165)	LGLFQSNLIQLSNIKIFQIFFFSYI
Oenothera elata subsp. Hookeri	(157)	LGLF---------------------
Oryza sativa	(157)	LGLF---------------------
Arabidopsis thaliana	(157)	LGLF---------------------
Spinacia oleracea	(157)	LGLF---------------------
Lactuca sativa	(157)	LGLF---------------------
Panax ginseng	(157)	LGLF---------------------
Populus trichocarpa	(157)	LGLFQIESI----------------
Zea mays	(157)	LGLF---------------------
Nicotiana tabacum	(157)	LGLF---------------------
Consensus	(165)	LGLF

Figure S3. Sequence alignment of PetD protein. Fully conserved residues are shaded in yellow, similar residues are shaded in green. The sequences from pea were aligned using MAFFT version 7 [1].

1. Katoh K, Standley DM: MAFFT Multiple Sequence Alignment Software Version 7: Improvements in Performance and Usability. Mol Biol Evol 2013, 30(4):772-780
